# Genomic insights of body plan transitions from bilateral to pentameral symmetry in Echinoderms

**DOI:** 10.1038/s42003-020-1091-1

**Published:** 2020-07-10

**Authors:** Yongxin Li, Akihito Omori, Rachel L. Flores, Sheri Satterfield, Christine Nguyen, Tatsuya Ota, Toko Tsurugaya, Tetsuro Ikuta, Kazuho Ikeo, Mani Kikuchi, Jason C. K. Leong, Adrian Reich, Meng Hao, Wenting Wan, Yang Dong, Yaondong Ren, Si Zhang, Tao Zeng, Masahiro Uesaka, Yui Uchida, Xueyan Li, Tomoko F. Shibata, Takahiro Bino, Kota Ogawa, Shuji Shigenobu, Mariko Kondo, Fayou Wang, Luonan Chen, Gary Wessel, Hidetoshi Saiga, R. Andrew Cameron, Brian Livingston, Cynthia Bradham, Wen Wang, Naoki Irie

**Affiliations:** 1 State Key Laboratory of Genetic Resources and Evolution, Kunming Institute of Zoology, Chinese Academy of Sciences, Kunming, China; 2 Sado Island Center for Ecological Sustainability, Niigata University, Niigata, Japan; 3 Dept. of Biological Sciences, California State Univesity, Long Beach, CA USA; 4 SOKENDAI, Kanagawa, Japan; 5 Urawa University, Saitama, Japan; 6 Japan Agency for Marine-Earth Science and Technology (JAMSTEC), Kanagawa, Japan; 7 Tokyo Metropolitan University, Yokosuka, Tokyo Japan; 8 Hokkaido University, Sapporo, Hokkaido Japan; 9 Dept. of Biological Sciences, Graduate School of Science, The University of Tokyo, Tokyo, Japan; 10 Providence Institute of Molecular Oogenesis, Brown University, Providence, RI USA; 11 Yunnan Agricultural University, Kunming, China; 12 Shanghai Institute of Biochemistry and Cell Biology, Center for Excellence in Molecular Cell Science, Chinese Academy of Sciences, Shanghai, China; 13 RIKEN Center for Biosystems Dynamics Research (BDR), Kobe, Hyogo Japan; 14 Universal Biology Institute, University of Tokyo, Tokyo, Japan; 15 NIBB Core Research Facilities, National Institute of Basic Biology, Okazaki, Aichi Japan; 16 Faculty of Social and Cultural Studies, Kyushu University, Fukuoka, Japan; 17 Key Laboratory of Systems Biology, Hangzhou Institute for Advanced Study, University of Chinese Academy of Sciences, Chinese Academy of Sciences, Hangzhou, China; 18 Chuo University, Tokyo, Japan; 19 Beckman Institute, Division of Biology and Biological Engineering, California Institute of Technology, Pasadena, CA USA; 20 Department of Biology, Boston Univerisity, Boston, MA USA; 21 School of Ecology and Environment, Northwestern Polytechnical University, Xi’an, China

**Keywords:** Evolutionary developmental biology, Evolutionary genetics

## Abstract

Echinoderms are an exceptional group of bilaterians that develop pentameral adult symmetry from a bilaterally symmetric larva. However, the genetic basis in evolution and development of this unique transformation remains to be clarified. Here we report newly sequenced genomes, developmental transcriptomes, and proteomes of diverse echinoderms including the green sea urchin (*L. variegatus*), a sea cucumber (*A. japonicus*), and with particular emphasis on a sister group of the earliest-diverged echinoderms, the feather star (*A. japonica*). We learned that the last common ancestor of echinoderms retained a well-organized Hox cluster reminiscent of the hemichordate, and had gene sets involved in endoskeleton development. Further, unlike in other animal groups, the most conserved developmental stages were not at the body plan establishing phase, and genes normally involved in bilaterality appear to function in pentameric axis development. These results enhance our understanding of the divergence of protostomes and deuterostomes almost 500 Mya.

## Abstract (web summary)

Li et al. investigate the evolution and genetic basis of the adult pentameral body plan in echinoderms using genomic, transcriptomic, and proteomic data. They determine that the last common ancestor of echinoderms contained an organized Hox cluster and endoskeleton genes, and suggest that cooption of bilateral development genes was involved in evolution of the pentameric body plan.

## Introduction

**Fig. 1:**
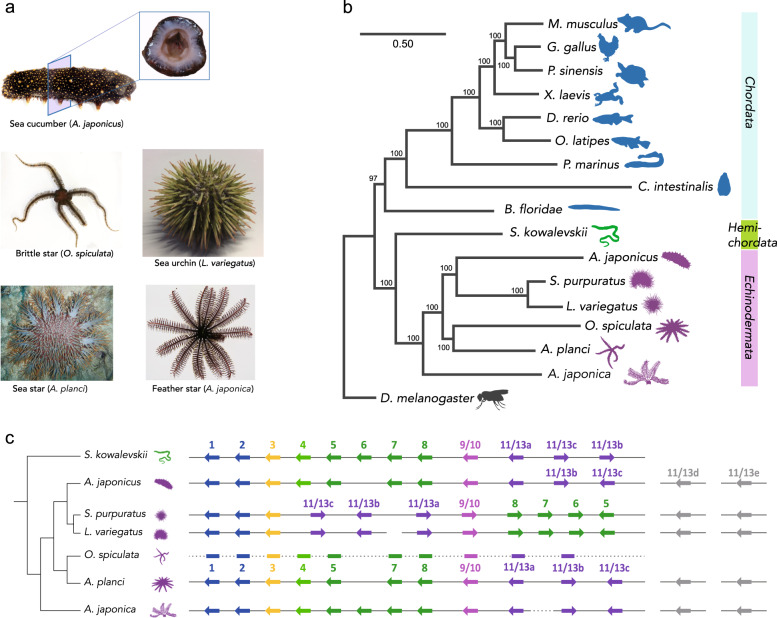
Echinoderms and their evolutionary diversity. **a** Echinoderm species of five living classes were analyzed in this study. Pentameral symmetry can also be observed in the transverse section of the sea cucumber (top), which otherwise shows apparent bilaterality. **b** Evolutionary rate and the phylogenetic tree constructed by RAxML software using the 1196 orthologous protein sequences identified by reciprocal best blast hit (RBBH). The values on branches represent bootstrap values. **c** Schematic representation of genomic organization of ambulacrarian Hox clusters. Arrows and horizontal lines represent Hox genes and chromosomal DNAs, respectively. Dashed lines indicate the presence of unconnected scaffolds. See Supplementary Fig. 13 for more detailed Hox cluster structures. Hox cluster structures of *S. kowalevskii*^9^*, A. japonicus*^56^, *S. purpuratus*^7^*, O. spiculata*^56^ and *A. planci*^8^ are according to the previous studies.

Bilateral symmetry is highly conserved throughout animal evolution. Echinoderms, a group closely related to chordates, are exceptional in this regard, developing pentameral symmetry as adults from bilaterally symmetric larvae. Even sea cucumbers, which show worm-like bilateral structures as adults, retain pentameral symmetry patterning along their oral-aboral axis (Fig. 1a)^1^. Understanding the development of pentameral symmetry would provide important insight into the evolutionary mechanisms of major structural changes in evolution^2,3^. To probe the genetic and developmental transitions behind the evolution of these unique echinoderm features, we sequenced genomes of the green sea urchin (*Lytechinus variegatus*) and the feather star (*Anneissia japonica*), representing nearly 500 Mya of evolutionary history. We also added developmental transcriptomic datasets for other echinoderms (Supplementary Tables 1–22 and “Methods”), such as the sea cucumber (*Apostichopus japonicus*), and proteome analyses of feather star skeleton to broadly analyze five living echinoderm classes. These data provide fundamental genomic, transcriptomic, and proteomic insights of body plan evolution in echinoderms, and enhance our understanding of the divergence of protostomes and deuterostomes.

## Results

### Genetic changes behind echinoderm evolution

The estimated genome sizes were 952 Mb for green sea urchin and 553 Mb for the feather star (Supplementary Fig. 1), with 30,238 and 26,838 protein-coding genes, respectively (Supplementary Tables 18–20 and 23). Basic features of these genomes such as GC content, gene length, and exon number were comparable to those of chordate and hemichordate species (Supplementary Fig. 2–7). Genome-wide analysis with 1196 one-to-one orthologs (Fig. 1b, Supplementary Fig. 7, and Supplementary Table 23–24) robustly corroborated recent reports^4,5^ that Echinodermata consists of the early diverged Crinoidea (including feather star), Asterozoa (including brittle star and sea star), and Echinozoa (including sea urchin and sea cucumber). This is consistent with paleontological evidence that suggests echinoderms first evolved with a stemmed, or imperforate extra-axial morphology^3^. We also found that the overall protein sequences of an early diverged echinoderm species, the feather star, showed a relatively low evolutionary rate. Meanwhile, echinoderms showed only slightly diverged protein sequences from vertebrates, as opposed to an ascidian, a species with highly derived morphological features, and with significantly diverged genomic sequences from vertebrates (Supplementary Fig. 8 and Supplementary Tables 26–28).

Given that echinoderms evolved unique features without significant genome-wide changes detected, we tested abundance in sets of gene families that may have played critical roles in the evolution of the echinoderm features. We first tested if numbers of genes potentially involved in development (such as genes involved in cell-cell communications) have expanded in the common ancestor of echinoderms (Supplementary Fig. 10). In contrast to our expectation, GO term enrichment analysis suggested that no such terms were enriched in the echinoderm-expanded gene set (Supplementary Fig. 11). Rather, GO terms such as “cell communication”, or “signal transduction”, were present in echinoderm-contracted genes (Supplementary Fig. 11a). Further, genes potentially involved in cytoskeletal regulation appear to have experienced extensive modifications during echinoderm evolution. For example, the “plectin repeat domain”, one of the important domains of cytolinkers that connect cytoskeletal elements with each other and to junctional complexes^6^, was not found in any of the five echinoderm species (Supplementary Fig. 12). Similarly, genes potentially involved in actin cytoskeleton regulation such as BCAR1/CAS and PIP5K were found to be positively selected during echinoderm evolution (Supplementary Table 29), suggesting substantial modifications of cytoskeletal function during echinoderm evolution.

### Hox clusters in ambulacrarians

We next analyzed genes in the Hox cluster, since previous studies implied that echinoderms may have undergone extensive changes to the genomic-arrangement of these genes early in their evolution^7^, but this is controversial^8^. By analyzing the echinoderm genomes, together with BAC sequencing and fluorescent in situ hybridization (FISH) experiments on feather star (see Supplementary Fig. 13), we found that *A. japonica* have 10 clustered Hox genes (Hox1 through Hox11/13a), together with two posterior genes (Hox11/13b and Hox11/13c) located with inverse directions more than 360 kb apart from the cluster of 10 Hox genes (Supplementary Fig. 14). This situation is reminiscent of hemichordate Hox gene clusters, which consist of 12 genes with the inversion/translocation of two posterior genes^9^. The consistent differences of Hox genes in feather star from those of hemichordates (*Saccoglossus kowalevskii* and *Ptychodera flava*^9^) were that the two posterior genes have face-to-face orientations, and two additional posterior genes (Hox11/13d and Hox11/13e) are present as reported in other echinoderm genomes^10^. These characteristics in turn suggest that the last common ancestor of echinoderms retained a canonical arrangement of 10 Hox genes, while its posterior genes had increased in number and changed their locations. This view accordingly indicates that the changes previously noted with the Hox gene clusters of echinoderms^7,8^, including the loss of Hox4 or Hox6 and the inversion/translocation of anterior genes, are lineage-specific events, and therefore are unlikely to be involved in the establishment of pentameral body plan. On the other hand, the involvement of the posterior Hox genes, are important candidates in the establishment of the pentameral body plan (Fig. 1c).

### Echinoderm embryogenesis show hourglass-like conservation

**Fig. 2:**
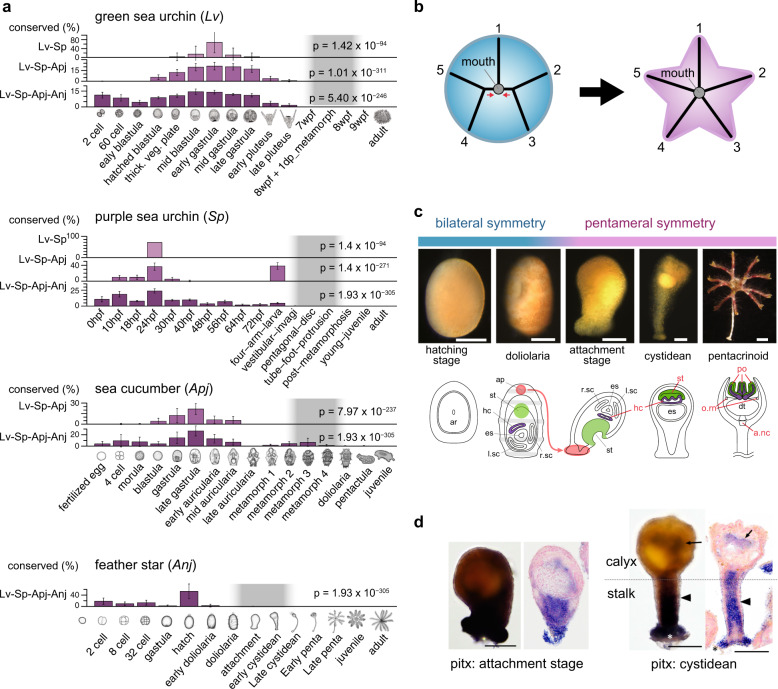
Evolutionarily conserved echinoderm stages and potential involvement of pitx signal in pentameral body plan development. **a** Mid-embryonic conservation found in echinoderm species. Based on expression distance (expDists, see also Supplementary Fig. 17) of orthologous groups (defined by orthomcl^57^), an evolutionary conservation of developmental stages were estimated for three taxonomic levels (Lv-Sp, Lv-Sp-Apj, Lv-Sp-Apj-Anj, see also “methods”). The vertical axis represents percentages of the stage being included in the most (top 1%) conserved stage-combinations^13^ (Ptop). Changes of the Ptop scores were significant among stages (Friedman test). Error bars represent S.D. of Ptop values. In each species, the developmental phase in which pentameral body plan establishment begins is colored in gray. **b** Possible evolutionary transition from bilateral symmetry to pentameral symmetric body plan suggested by paleontological studies^61^. The basal echinoderms had a bilaterally symmetric ambulacral system that is arranged in a 2–1–2 pattern (left); consisting of one unpaired ambulacrum (1) and two ambulacra with a distal bifurcation (2&3, 4&5) and a single unpaired ambulacrum (1). **c** Feather star development from bilateral symmetry to pentameral symmetry. a.nc aboral nerve center, ar archenteron, ap adhesive pit, dt digestive tract, es enteric sac, hc hydrocoel, l.sc left somatocoel, o.rn oral ring nerve, po podia, r.sc right somatocoel, st stomodeum. **d** *pitx* gene expression detected in embryos of attachment stage and cystidean stage. In cystidean embryos, *pitx* was expressed in the tissues around the gut (arrows) and the inner tissue of the whole stalk (arrowheads). Scale bars: 100 μm. The expression was detected by in situ hybridization with whole mount (left) and sectioned specimens (right).

We next tested if evolution of echinoderm embryogenesis follows the developmental hourglass model^11^ as supported in several animal groups^12–16^. The phylotype hypothesis of the hourglass model predicts that anatomical features of most conserved-embryonic phase represent the body plan of their animal phylum^11,17,18^. We tested if the developmental stages most critical for the pentameral body plan show the highest transcriptomic conservation. Recent studies reported that the transcriptomic conservation is instead around blastula to gastrula in sea urchins^19,20^, however, these studies only examined embryonic sea urchins. We thus analyzed gene expression profiles by covering early-to-late embryos of four diverse echinoderm species, including the publicly available data of purple sea urchins^21,22^ and a sea cucumber^23^ (Supplementary Figs. 15 and 16). Unexpectedly, while the hourglass-like conservation was observed, the most conserved phase was not bracketing the pentameral establishing stages (defined as stages when first pentameral symmetric structures appear), but it was instead during gastrulation (Fig. 2a and Supplementary Figs. 17–18). This mismatch between the most conserved phase and the phase for establishing the body plan was also supported by pair-wise comparisons of one-to-one orthologs (Supplementary Fig. 19). These results suggested that, unlike in other animal groups such as vertebrates, the phase for establishing the body plan in echinoderms has experienced substantial diversification during their evolution, further implying that the phylotype hypothesis may not fit within echinoderm embryogenesis. However, a potential caveat of this conclusion would be that the conservation signals from adult rudiments could have been obscured by larval tissues, as adult rudiments share only a small proportion of embryo in early metamorphic stages. Similar analyses with dissected adult rudiment, or single-cell RNAseq technology could clarify this point. While possible contribution of pleiotropic constraints were supported between the closely related species as reported previously^24,25^ (Supplementary Figs. 20 and 21), further studies are needed to clarify the evolutionary mechanism behind the unique evolution of echinoderm embryos.

### Partial co-option in pentameral body plan establishment

Paleontological studies suggested the possible evolution of pentameral body axes through changes in the mechanisms of bilateral symmetry^26^ (Fig. 2b). However, developmental genes that control the pentameral symmetry remains largely unknown. We thus focused on genes that are involved in Left/Right-patterning and other axis-forming in bilaterians^27–31^ and examined expression patterns of their homologs in the feather star, particularly at the attachment and cystidean stages when the pentameral body plan forms (Supplementary Figs. 22 and 23). Among the genes examined, *pitx* exhibited relatively strong expression in the calyx, where the pentameral structure first becomes evident (Fig. 2c–d,). Weak expression of *chordin* was also detected in the calyx (Supplementary Fig. 23). Meanwhile, expression of *bmp2/4*, *nodal*, *lefty* and *not* were detected most during gastrula to doliolaria stages (Supplementary Fig. 22), but not in the calyx (Supplementary Fig. 23), implying that these genes are possibly involved in the body patterning during bilateral planktonic development rather than pentameral body plan development. These results suggest that evolution of the pentameral body plan may have associated partial co-option of genes involved in existing body axes, which in part, coincides with paleontological predictions that modification of bilateral patterning system contributed to the pentameral body plan establishment^26^.

### Proteome analyses of echinoderm skeleton

**Fig. 3:**
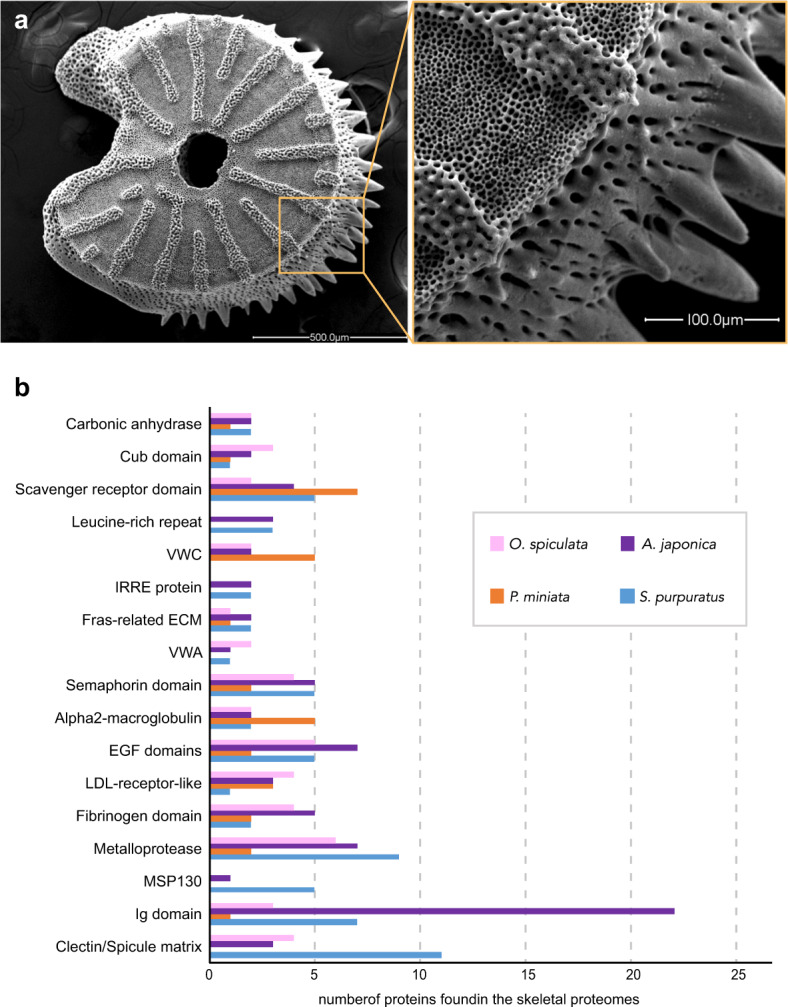
Skeletal element related proteins/domains identified in echinoderms. **a** SEM image of a skeletal element isolated from the feather star (*A. japonica*). **b** Proteins present in the feather star skeletal proteome were isolated from adult skeleton and identified by comparison of LC/MS/MS data to the genes computationally identified in the feather star genome. These proteins were compared to those found in skeletal proteomes of the sea urchin *S. purpuratus*^62–64^, the sea star *P. miniata*^36^ and the brittle star *O. spicullata*^35^. The most prevalent proteins are shown in the figure, along with the number of different proteins from the listed groups present in each species’ proteome. The feather star skeletal proteome contains members of each of the protein families shown. The other echinoderm species are missing some of these proteins in their skeletal proteome and the number of members in each protein family varies between groups.

Mineralized endoskeleton is another notable feature of echinoderms. Despite the widely conserved endoskeleton structures in echinoderms, some of the proteins first identified in sea urchin biomineralization, such as MSP130, have not been identified in the skeleton of other echinoderm species. The MSP130 gene was suggested to have originated in prokaryotes and was introduced into metazoan genomes, including echinoderms, by horizontal gene transfer^32^. An MSP130-like gene involved in biomineralization has also been identified in a polychaete^33^. The authors suggest that the MSP130 gene was present in the common ancestor to bilaterians, rather than being introduced into protostomes and deuterostomes in separate lateral transfer events. The MSP130 protein was then co-opted into skeleton formation at some point in echinoderm evolution. The gene was duplicated in sea urchins and the resulting paralogues acquired repetitive regions^32^. The MSP130 gene is present in other echinoderm genomes, but is not utilized in the proteome of brittle stars^34,35^ or sea stars^36^. Here, we performed a proteome analysis and identified 280 proteins that are included within the mineral of the adult feather star skeleton (see “Methods”). These skeletal proteins included a protein similar to the urchin MSP130 proteins, suggesting that the ancestral echinoderm had co-opted this single protein into biomineralization (Supplementary Fig. 24). A number of other genes encoding skeletal proteins and domains conserved between the purple sea urchin (*S. purpuratus*) and the feather star were also identified (Fig. 3). Among these, we found two proteins with C-type lectin domains, which are also found on the urchin spicule matrix proteins. C-type lectin proteins are absent in sea star skeletons^36^, and present in only a few copies in brittle star skeletons^34,35^. Sea urchin skeletons utilize a large number of C-type lectins, mostly with repetitive stretches of acidic amino acids^37^, while the feather star and brittle star proteins lack these repetitive domains (Supplementary Fig. 25). A possible evolutionary scenario to explain these differences is that the ancestral skeletal C-type lectin genes experienced extensive duplication and acquisition of repetitive domains in the sea urchin lineage. The use of C-type lectins in the sea star skeleton was lost, while the C-type lectins in the feather star and brittle star skeletal proteomes remain largely unchanged. Together, these results suggest that the precursors to all of the genes and domains used in echinoderm skeleton were already present in the common ancestor to echinoderms, which emerged 589.7 Mya (Supplementary Fig. 7). Additionally, these skeleton-related genes may have undergone frequent duplication and loss in specific lineages, together with frequent changes in gene expression, since expression of MSP130-like genes and C-type lectin genes in the skeleton forming cells were lost in some lineages, even though these genes exist in their genomes. In summary, our study highlights the genomic, transcriptomic and proteomic changes behind the evolution of unique features in echinoderms, and offers an exceptional case in understanding the general tendency for the evolution of body plans.

## Methods

### Animal care and use

Animal care and experimental procedures and were conducted in strict accordance with guidelines approved by the Animal Experiments Committee of University of Tokyo (approval ID: 14–03, 16–2). All efforts were made to minimize suffering. Individual animals and embryos were selected blindly from wild types.

### DNA extraction, library construction, and genome sequencing

*Lytechinus variegatus*: Genomic DNA was extracted from sperms from a single male. We first constructed five different short-insert libraries (394, 424, 479, 496, and 522 bp. See also Supplementary Table 1) from the genomic DNA samples and sequenced them using the Illumina HiSeq 4000 system to survey the genome complexity. After obtaining the genome size, we further constructed four mate-pair libraries (2–18 Kb. See also Supplementary Table 1) from the same DNA sample, and sequenced them for further assembly. The DNA and genome we obtained in this project is independent from those available through EchinoBase (*Lytechinus variegatus* genome v.2.2).

*Anneissia japonica*: After collecting adult feather stars in the cove of Koajiro, Sagami Bay (Misaki, Japan) by scuba diving, sperms from a single male was collected during the breeding season when the gonads were ripe with mature gametes. Sperms were embedded in ~0.5% low-melting agarose plugs (SeaPlaque GTG Agarose, Lonza), and in-gel digestion of proteins was performed by immersing the plugs in digestion buffer (10 mM Tris-Cl pH 7.5, 50 mM NaCl, 10 mM EDTA, 0.5% SDS, 200 mg/mL Proteinase K) at 55 °C, overnight. The gel-plugs were washed repeatedly with TE buffer and stored in TE at 4 °C until use. DNA was released from the gel-plugs using GELase (Epicenter). The DNA was further purified using QIAGEN Genomic-tip 20/G (QIAGEN) and dissolved in TE. Five different short-insert libraries (277, 324, 381, 450, and 477 bp) were constructed and sequenced with Illumina HiSeq 4000 system to survey the genome complexity. After obtaining the genome size, we further constructed six mate-pair libraries (2–18 Kb) and sequenced them for further assembly (Supplementary Table 1).

### K-mer-based estimation of genome sizes

We first compared the performances in genome size estimation by K-mer frequency method and GenomeScope. The genome size of green sea urchin estimated by GenomeScope was about 650 Mbp, while that of kmerfreq method was about 952 Mbp. Considering that the genome size estimated by kmerfreq was closer to the genome size estimated from *C*-value (0.92, www.genomesize.com) than GenomeScope, we decided to apply kmerfreq method for the genome size estimation. Following formula was used for estimating genome size: *Genome* size (bp) = K-mer number/*average* depth *of K-mer*. Based on the rate of occurrence of K-mers in each genome, the read depths for feather star and green sea urchin were estimated as 147 and 124, respectively, leading to genome size estimations of approximately 553 Mb for feather star and 952 Mb for green sea urchin (Supplementary Fig. 1).

### Raw read filtering and error correction of short-read libraries

HiSeq raw reads with the following features were regarded as low-quality reads and were filtered out: [1] Reads containing >10 bp adapter sequences; [2] Reads in the small insert libraries (refer to Supplementary Tables 1 and 2) having >10 bp overlap; [3] Reads having *N*’s >10% of their length; [4] PCR duplicates (paired-end reads completely identical); [5] Reads containing >40 bp low-quality (phred quality score ≤ 5) bases. After the filtering process, we further corrected the qualified K-mers. In brief, K-mers with sequencing errors are usually low in frequency, and we thus corrected these K-mer sequences by refering to high-frequent reads. If the erroneous sites could not be corrected, the low-frequency K-mers from the reads were trimmed. No error correction was made for the long-insert libraries (refer to Supplementary Tables 1 and 2), as these were only used for scaffolding. The SOAPec_bin_v2.03 software was used to correct the error within reads. Command line: SOAPec_bin_v2.03/bin/KmerFreq_AR -q 33 -b 100000000000 -k 17 -p output reads_files_list; SOAPec_bin_v2.03/bin/Corrector_AR -Q 33 -k 17 output.freq.cz output.freq.cz.len reads_files_list.

### Gene set and genomes obtained from public database

Refer to Supplementary Table 24 for the publicly available gene set and genomes used in this project.

### Genome assembly

Genome sequences with the filtered and/or corrected data were assembled by Platanus software. The assembly was carried out using the following steps: (a) Contig construction: Reads from short-insert (<1 Kb) libraries were split into K-mers and used to construct a de Bruijn graph. Short branches caused by errors were removed by “tip removal” step and short repeats were resolved by K-mer extension. Bubble structures caused by heterozygosity or errors were removed. At last, subgraphs without any junctions represent the contigs. (b) Scaffold construction: All the filtered clean reads were re-aligned onto the contig sequences, and the scaffolds were constructed by weighting the consistent rate and paired-end reads relationships on the contigs. Heterozygous regions were removed as bubble or branch structures on the graph by the “bubble removal” or “branch cut” step. These simplification steps are characteristic of Platanus and especially effective for assembling complex heterozygous regions. (c) Gap filling: Paired-end reads have one end mapped on the contig with the other end located in the gap region were used to fill the gaps in the genome assembly by GapCloser1.10 software. Then the very short assembly sequences (contig shorter than 500 bp) were removed in the genome assembly. The detailed command lines of the Platanus assembly were shown as below: Feather star: platanus assemble -o contig.fa -f short_clean_reads.fq -k 69 -u 0.2 -m 200; platanus scaffold -c contig.fa -b contigBubble.fa -o scaffold.fa -IP R1.fq R2.fq -OP R1.fq R2.fq -u 0.2. Green sea urchin: platanus assemble -o contig.fa -f short_clean_reads.fq -k 29 -u 0.3 -m 200; platanus scaffold -c contig.fa -b contigBubble.fa -o scaffold.fa -IP R1.fq R2.fq -OP R1.fq R2.fq -u 0.3. Results by K-mer analysis Supplementary Fig. 1, and statististics of feather star and green sea urchin genomes are shown in Supplementary Tables 3 and 4.

### Assessment of assembled genomes

The completeness of the feather star and green sea urchin assemblies was assessed by the BUSCO program (version 2.0), using the eukaryotic and metazoan libraries (Supplementary Tables 6–8). Reads from the short-inserts libraries were also mapped to these assembled genomes by BWA and SAMtools software (bwa index -a bwtsw genome.fa; bwa aln -t 6 genome.fa reads.fq; samtools view -b -S out.sam > out.bam; samtools flagstat out.bam) to assess the genomic quality (Supplementary Table 9). In addition, coverage ratio of de novo assembled transcripts obtained by Trinity <ver. 2.2.0 > (perl Trinity --JM 200 G --seqType fq --left reads_R1.fq --right reads_R2.fq --SS_lib_type FR -output out) and TGICL software (tgicl -F transcripts.fasta) over the sequenced genomes using BLAT software (blat genome.fa transcript.fa -t = dna -q = rna out.psl) (Supplementary Tables 10–13. De novo assembled the transcripts (made by the RNAseq data we obtained for each species) were also aligned to the fileterd genome and confirmed that 98.64% transcripts in feather star and 99.53% transcripts in green sea urchin were aligned.

### GC content of genome

GC content of the feather star and green sea urchin genomes were estimated using a sliding window approach. Briefly, a 500 bp sliding window (250 bp stepwise) was employed to scan along the genome and calculate the GC content, and found that the average GC content of feather star and green sea urchin is about 33.22% and 33.71%, respectively. Both of these values were found to be similar with those of hemichordate and most chordate species except lamprey (Supplementary Fig. 2).

### Repeat annotation

Tandem repeats in the genomes were identified using Tandem Repeat Finder^38^ (v4.04 http://tandem.bu.edu/trf/trf.html) with default parameters (trf sequence.txt 2 7 7 80 10 50 2000 -d -h, these number means: Match, Mismatch, Delta, PM, PI, Minscore, and MaxPeriod), and non-interspersed repeats in the genome using RepeatMasker^38^ (open-4–0–5) with default parameters (-nolow (Not mask low_complexity DNA or simple repeats) -no_is (Skips bacterial insertion element check) -norna (Does not mask small RNA (pseudo) genes) -parallel 1 (The number of processors to use in parallel)). Transposable elements (TEs) were identified on both the DNA and protein levels. On the DNA level, RepeatModeler (v1.0.4) and RepeatScout^39^ (version 1.0.5) was used to build repeat libraries. In feather star, LTR_FINDER^40^ (v1.0.5) software were additionally used to build de novo repeat libraries. RepeatMasker was performed on both de novo libraries and repbase (RepBase16.02) separately to identify homologous repeats with default parameters with format set with 2 (-w 2-table). On the protein level, RM-BLASTX within RepeatProteinMask was used to query the TE protein database with -noLowSimple and *P*-value 0.0001 (Supplementary Fig. 3 and Supplementary Tables 14–17).

### Prediction of protein-coding genes

Prediction of protein-coding genes was based on integration of three different methods, namely, ab initio prediction, homology-based annotation and RNAseq-based annotation. For ab initio prediction, Augustus^41^ (v2.5.5, --uniqueGeneId=true [output gene identifyers] --noInFrameStop=true [Do not report transcripts with in-frame stop codons] --gff3=on [output in gff3 format] —strand=both [--strand=forward and --strand=backward]) and GENSCAN^42,43^ (v1.0, -mini_cds 150 -cds_ns 10) software were used to predict genes. In feather star, SNAP^44^ (using ** species for gene prediction) and GlimmerHMM^45^ (v3.02, using ** species for gene prediction, and -f [Do not make partial gene predictions] -g [Print output in gff format]) softwares were also used in this analysis. These four software programs were trained by using lamprey, human, ciona, and zebrafish, respectively. Short genes (CDS length < 150 bp) and low-quality genes (gaps covered more than 10% of the coding region) were discarded. Proteins from human (Ensembl:GRCh38), mouse (Ensembl: GRCm38), chicken (Ensembl: Gallus_gallus-5.0), green anole lizard (Ensembl: AnoCar2.0), Xenopus tropicalis (Ensembl: JGI_4.2), zebrafish (Ensembl: GRCz10), sea lamprey (Ensembl: Pmarinus_7.0), lancelet (LanceletDB: v18h27.r3_ref), *Ciona instestinalis* (NCBI: GCA_000224145.1), acorn worm (NCBI: GCF_000003605.2) and purple sea urchin (NCBI: GCF_000002235.4) were used in the homology-based annotation using tblastn with *e*-value 1e-5. Blast hits that correspond to reference proteins were concatenated by Solar software and low-quality records were filtered out. Sequence of each reference protein was extended to upstream and downstream by 2 Kb to represent the protein-coding region with default parameters. GeneWise software was used to predict gene structure contained in each protein-coding region. For each gene locus, the longest coding region and/or highest genewise score was retained. In RNAseq-based method, the coding sequences defined by transcripts was aligned against the genome by BLAT^46^ (v34, identity > 90%, coverage > 90%), thereby defining the splicing orientation of coding region. Then, PASA software was used to link the spliced alignments with default parameters. The EvidenceModeler^47^ (EVM, ver. 1.1) software was further used to integrate data derived from the three methods into an EVM-derived gene set with default parameters, the weight of de novo, homolog and complementary DNA (cDNA) are 1, 5, and 10. Finally, 26,838 and 30,238 protein-coding gene models were annotated in feather star and green sea urchin genome, respectively (Supplementary Table 18).

### Annotation of gene function, non-protein-coding genes

InterProScan (v4.8) was used to screen these genes’ protein sequences against five databases (including: Pfam, release 27.0, prints, release 42.0, prosite, release 20.97, ProDom, 2006.1, and smart, release 6.2) to determine the InterPro and GO number of those predicted protein-coding genes. In addition, KEGG, COG, NR, Uniprot/SwissProt, and UniProt/TrEMBL databases were searched for homology-based functions (Supplementary Tables 19 and 20) using blastp (v2.2.26) with *e*-value (1e-5). For non-coding genes, tRNAscan-SE 53 (v1.3) software for eukaryotes was used for tRNA annotation in the genomic assembly with default parameters. Ribosomal RNA (rRNA) annotation was based on homology information of invertebrate rRNA collections using BLASTN (v2.2.26) with *e*-value set as 1e-5. The small nuclear RNA (snRNA) and microRNA (miRNA) were predicted by INFERNAL software (v0.81) against the Rfam database (Release 9.1) with default parameters. The statistical results are shown in Supplementary Tables 21 and 22.

Potential functions of protein-coding genes were predicted using InterProScan^48^ (v4.5), against five databases (Pfam, release 27.0, PRINTS, release 42.0, PROSITE, release 20.97, ProDom, 2006.1, and SMART, release 6.2). In addition, KEGG, COG, NR, Uniprot/SwissProt and UniProt/TrEMBL databases were searched for homology-based functions (Supplementary Tables 19 and 20). For non-coding genes, the tRNAscan-SE^49^ (v1.3) software for eukaryote was used for tRNA annotation in the genomic assembly. rRNA annotation was based on homology information of invertebrate rRNA collections using BLASTN (v2.2.26) with *e*-value (1e-5). The snRNA and miRNA were predicted by INFERNAL software (v0.81) against the Rfam database (Release 9.1). The statistical results are shown in Supplementary Tables 21 and 22.

### Gene family analysis

*orthoMCL*: orthoMCL^50^ was used to find ortholog genes and/or gene families (ortholog groups) among different species. Amphioxus (*Branchiostoma floridae*), zebrafish (*Danio rerio*), ciona (*Ciona intestinalis*), Drosophila (*Drosophila melanogaster*), chicken (*Gallus gallus*), acorn worm (*Saccoglossus kowalevskii*), green sea urchin (*Lytechinus variegatus* [Lv]), purple sea urchin (*Strongylocentrotus purpuratus* [Sp]), medaka (*Oryzias latipes*), mouse (*Mus musculus*), brittle star (*Ophiothrix spiculata*), feather star (*Anneissia japonica* [Anj]), lamprey (*Petromyzon marinus*), sea cucumber (*Apostichopus japonicus* [Apj]), sea star (*Acanthaster planci*), frog (*Xenopus laevis*), turtle (*Pelodiscus sinensis*) gene set were prepared and used here. Gene families and ortholog genes identified by this OrthoMCL is shown in Supplementary Figs. 5 and 6. For ortholog groups identified among echinoderms were 15618 for Lv-Sp-Apj-Anj, 14758 for Lv-Apj-Anj, 15035 for Lv-Sp-Apj, 14231 for Lv-Sp and 13649 for Lv-Anj.

*Reciprocal best blast hit (RBBH), 1:1 orthologs*: We also analyzed the ortholog genes by RBBH method. We first selected feather star as the reference species, and aligned the protein sequences in all other 16 species to feather star gene set and vice versa by blast. Second, the aligned results were filtered by *e*-value (1e-5) and retained only the reciprocal best blast hit for each gene-gene pairs. Third, orthologous gene pairs in all of the 16 species were extracted. Finally, we identified 1196 ortholog genes among these 17 species.

### Phylogenetic tree construction and divergence time

*Molecular phylogenetic analysis*: 1196 RBBH ortholog genes (1,447,456 aa) in each species were combined into a super-gene in the same gene order, followed by phylogenetic analysis using RaxML^51^ (with PROTGAMMAAUTO model, *Drosophila melanogaster* was used as the outgroup species) through these super-genes (Fig. 1b and Supplementary Table 25). Both of the reconstructed phylogenetic trees robustly showed three clusters, including echinoderms, acorm worm, and chordates. Among them, feather star was the earliest diverging species in echinoderms, brittle star and sea star form one branch, sea urchin and sea cucumber form another branch.

*Divergence time estimation*: To estimate the divergence time, the super-genes prepared above were analyzed by MCMCtree software, together with several calibration points downloaded from TimeTree website (http://www.timetree.orgs) (Supplementary Fig. 7).

### Relative evolutionary rates of species

To determine the relatively evolutionary rates of echinoderm species, the super-genes, which we produced from 1196 RBBH orthologs were used. LINTRE software and RRT (Tajima’s relative rate test) analysis were employed, and *Drosophila* was used as an outgroup to determine the root of the whole tree (Supplementary Fig. 8 and Supplementary Tables 26–28. In addition to the LINTRE analysis, R-package “APE” was also used to deduce robust conclusion (Supplementary Fig. 9).

### Expansion and contraction of gene families

To identify expanded and contracted gene families in the common ancestor of echinoderms, the gene family result generated from OrthoMCL were used and analyzed by CAFE software (Supplementary Fig. 11). Profiles of GO terms, protein domains and KEGG pathways of these expanded and contracted genes are shown in Extended data 1 (Extended_data1.xlsx).

### Domains lost in echinoderms

Domains found in any of the chordate species, but not found in any of the the echinoderm species were defined as domains lost in echinoderm lineage. Six echinoderm species (*Apostichopus japonicus*, *Lytechinus variegatus*, *Acanthaster planci*, *Ophiothrix spiculata*, *Anneissia japonica*, and *Strongylocentrotus purpuratus*) and nine chordate species (*Mus musculus*, *Branchiostoma floridae*, *Ciona intestinalis*, *Petromyzon marinus*, *Oryzias latipes*, *Gallus gallus*, *Xenopus laevis*, *Pelodiscus sinensis*, and *Danio rerio*) were blasted (>50% identity and >30% align ratio) to the acorn worm (*Saccoglossus kowalevskii*) protein gene set and searched for potential domains lost in echinoderms. Seven-hundred forty-seven genes were identified to be the lost genes in echinoderms. Among these genes, six genes were not found in any of the nine chordate species, but found in acorn worm. These genes were enriched with GO terms of biosynthetic process, metabolism process, and the establishment of localization.

### Hox cluster analysis

*Cloning of Hox genes*: To further confirm sequences of Hox genes in the feather star, a total of nine hox genes had been cloned from *Anneissia japonica* using RT-PCR (Tsurugaya et al., in preparation).

*Preparation of A. japonica BAC library*: We constructed BAC library, using genomic DNA prepared from the male gonadal pinnules that contained testes. The DNA was partially digested with the restriction enzyme MboI, size-fractionated, and cloned into the vector pCCBAC1(EPICENTER). The bacterial strain DH10B T1 phage resistant (Invitrogen) was used for transfecting the BACs for constructing the library. Single clones were picked into 384-well plates and preserved. Two batches of libraries were produced, which were named Oj1 (average insert size ~100 kb, 35,712 clones) and Oj2 (average insert size ~78 kb, 45,977 clones).

*Screening and cloning of BAC clones containing hox genes*: Using the cDNA fragments of nine hox genes (hox1, hox2, hox4, hox5, hox7, hox8, hox9/10, hox11/13a, and hox11/13c. Tsurugaya et al., in preparation), we screened the BAC library of *A. japonica* for the clones that contained Hox genes and their neighboring regions. This screening yielded 23 clones in total, which, however, were not contiguous but separated into four groups.

*FISH analysis*: Probes for FISH were derived from clones out of the *A. japonica* BAC library. BAC clones used for FISH were Oj1–26E10 (containing hox1), Oj2–17D15 (hox2), Oj2–75D03 (hox4 and hox5), Oj2–78N14 (hox7 and hox8), Oj1–50I03 (hox8 and hox9/10), and Oj2–102A05 (hox11/13c). BAC clone DNAs were isolated using Qiagen Plasmid Midi Kit (Qiagen) and labeled with biotin or digoxigenin by using Nick Translation Kit (Roche). Hybridization mix was prepared as described previously (3). Two color-chromosomal FISH was carried out as described previously (1, 2) with the following modifications. Blastula or early gastrula stage embryos were treated with 0.08% colchicine (Sigma) in sea water for 30 min. Embryos were fixed in methanol glacial acetic acid (3:1) fixative at 4 °C overnight, then transferred to 100% ethanol, and stored at –20 °C. To prepare metaphase spreads, 80 µL of 60% acetic acid was added to a microfuge tube containing 50–100 embryos. Three minutes later, embryos were dropped onto a prewarmed (48 °C) slide glass, and left until dry (about 30 min). Before hybridization, the slides were treated with 0.5% pepsin (1:100, Wako) in 0.01 N HCl for 3 min, and washed in phosphate-buffered saline (PBS) three times. Then the slides were post-fixed in 1% paraformaldehyde in PBS at r.t. for 30 min, and washed in PBS twice. After dehydration, the air-dried slides were treated with acetone at r.t. for 10 min, and dried again. Following the denaturation of chromosomal DNA and dehydration, hybridization was carried out at 43 °C for 16 h. FISH images were taken using an Olympus BX60 microscope equipped with an Olympus DP70 camera.

*Identification of clustered Hox genes*: In scaffold 288292 (about 1.86 Mbp in length), hox1, hox2, hox3, hox4, hox5, hox6, hox7, hox8, hox9/10, and hox11/13a were identified. The ten Hox genes were aligned in the order, spanning about 480 kb in length, with the 3ʹend of hox1 about 392 kb away from the end of the scaffold. In scaffold 287987 (about 96 kb), hox11/13b and hox11/13c were identified. To see whether the 12 Hox genes form a single cluster, we carried out two color-chromosomal FISH, using the BAC clones (described above) as probes. The FISH analysis revealed that the eight genes contained in the BAC clones were in close vicinity to one another on a single chromosome (comprising of two sister chromatids). However, the gene order or relative positions of the two scaffolds on the chromosome could not be clarified, leaving four possible gene orders of 12 Hox genes undetermined. Thus, it is suggested that 12 Hox genes are present on a single chromosome, forming two subclusters separated by at least 400 kb in the genome of *Anneissia japonica*^10^, hox11/13d and hox11/13e, in another scaffolds 2266 and 6788, respectively. This suggests that the two genes are localized apart from the subcluster of ten Hox genes, which situation is consistent with the previous report showing that Hox11/13d and Hox11/13e do not reside in the Hox gene cluster in echinoderm genomes^10^.

### Embryo collection and RNA extraction

*Lytechinus variegatus*: Adult green sea urchins were originally obtained from Reeftopia in Florida (FL) or from the Duke Marine lab in Beaufort NC. *L. variegatus* total RNA was prepared from wild type embryos per timepoint using TRIzol (Invitrogen) and DNase treatment. RNA quantitation and integrity were determined using a Qubit® 2.0 Fluorometer (Life Technologies) and a 2100 Bioanalyzer (Agilent Technologies). Total RNA was subjected to three iterations of polyA selection using Dynabeads (Life Technologies) prior to cDNA synthesis. Following stages were collected for RNA extraction and fixation; 2 cell (1 h post fertilization), 60 cell (2.5 hpf), EB (Early Blastula, 4 hpf), HB (Hatched Blastula, 7 hpf), TVP (Thickened Vegetal Plate, 10 hpf), MB (Mesenchyme Blastula, 12 hpf), EG (Early Gastrula, 13 hpf), MG (Mid Gastrula, 15 hpf), LG (Late Gastrula,18 hpf), EP (Early Pluteus, 36 hpf), LP (Late Pluteus, 48 hpf), 7 wpf (7 weeks post fertilization), 8 wpf (8 weeks post fertilization), 1 day post metamorphosis, 1 week post metamorphosis, and adult. In addition, RNA from larval region of 8 weeks post fertilization (8 wpf Larva), and rudiment region of 8 weeks post fertilization (8 wpf Rudiment) were also extracted by dissecting the 8 wpf embryo. Results based on analyses with RNAseq data from two cell to Late pluteus were published^52^. Two independent biological samples were prepared for all the sampled stages.

*Apostichopus japonicus*: Embryos of fertilized eggs, 4 cell (2 h post fertilization (hpf)), morula (6 hpf), blastula (14 hpf), gastrula (29 hpf), late gastrula (34 hpf), early auricularia larva (48 hpf), mid-auricularia larva (69 hpf), late auricularia larva (15 days post fertilization (dpf)), metamorphosis 1–4 (17–19 dpf), doliolaria larva (19 dpf), pentactula larva (27 dpf), and juvenile (51 dpf) stage were collected and used for this study, as previously described^23^. Three independent biological samples were prepared for all the sampled stages.

*Anneissia japonica*: Adult *Anneissia japonica* (previously called as *Oxycomanthus japonicus*, see Summers et al.^53^ for the nomenclature) were collected from rocky substrate of about 10 m depth at Koajiro, Sagami Bay, and kept in the sea until the day of spawning. Spawning check was carried out at every neap tide days during October and November, 2015. Spawning was observed in the evening of 20th and 21st of October, 2015, and seven females spawned in total. The obtained eggs were very fragile and surrounded by mucus. Small amounts of the spawned unfertilized eggs were separated in 1.5 mL tubes (100 μL each) for RNA extraction and fixation. The rest of the eggs were inseminated immediately by diluting concentrated sperm, which were directly collected from genital pinnules. The fertilized eggs were washed with filtered sea water several times to remove the mucous, and separated in the filtered sea water in plastic vessels for culture. The culture was done at room temperature (about 10–20 °C). Following stages were collected for RNA extraction and fixation (Supplementary Fig. 15); 2 cells (1.5 h post fertilization), 8 cells (2.5 hpf), 32 cells (3.5 hpf), gastrula (8 hpf), hatching stage (17 hpf), early doliolaria (24 hpf), mid-late doliolaria (36 hpf), attachment stage (3–4 days pf), early cystidean (4–7 days pf), late cystidean (7–9 days pf), early pentacrinoid (3 weeks pf), late pentacrinoid (1.5 months pf), juvenile (2.5 months pf), arm branching stage (6–7 months pf), and adult (9 months pf). For the RNA extraction, more than 50 μL of specimens were diluted in the 10x volume of TRIzol reagent (Invitrogen). The tissue of the specimens were destructed by pipetting with a micro syringe or grinding with a pestle and mortar in the TRIzol reagent, and immediately stored in –80 °C. For the fixation, specimens were fixed with 4% paraformaldehyde in 0.5 M NaCl and 0.1 M 3-(N-morpholino) propanesulfonic acid (MOPS), pH 7.0 for several days at room temperature (about 22 °C). Fixed specimens were washed with 70% ethanol three times, and stored in 70% ethanol at –20 °C. Two independent biological samples were prepared for all the sampled stages.

### RNA sequencing and gene expression data

After adjusting total RNA amounts between samples, non-stranded sequencing libraries (with the TruSeq protocol) were constructed and sequenced using the Illumina HiSeq 4000 platform. For the sea cucumber (*A. japonicus*) samples, Quartz-seq amplified libraries were made as previously described^13^. Qualities of raw reads were evaluated using FastQC program (http://www.bioinformatics.bbsrc.ac.uk/projects/fastqc/). Read length and single/paired information are as follows; *L. variegatus* (100 bp, paired-end), *A. japonica* (150 bp, paired-end), *A. japonicus* (100 bp, single-end). Adapter sequences of Quartz-Seq samples (Mm early stages 2-cell-blastocyst) were removed using the fastq-mcf program (https://code.google.com/p/ea-utils/wiki/FastqMcf) as previously described^13^. RNAseq data were then mapped to genomes of each species using HISAT2 program^54^ (ver. 2.05), and calculated relative expression levels by StringTie^55^ (ver. 1.3.5) with species-specific GTF files.

*Apostichopus japonicus*: For the gene expression levels of Japanese sea cucmber, genome and GTF files reported by Zhang et al.^56^ were used.

*Strongylocentrotus purpuratus*: Developmental transcriptomes of the purple sea urchin was obtained from SRA (Accession: PRJNA81157), sequenced by Tu et al.^21,22^. The dataset contained developmental stages of 0 hpf, 10 hpf, 18 hpf, 24 hpf, 30 hpf, 40 hpf, 48 hpf, 56 hpf, 64 hpf, 72 hpf, four arm larva, vestibular invagi, pentagonal disc, tube foot protrusion, post metamorphosis, young juvenile, adult.

### Identification of conserved stages

Whole embryonic, comparative transcriptomic analysis was performed as previously described to find evolutionarily conserved developmental stages^13^. Relative expression levels (TPM) of ortholog groups (defined by orthomcl^57^) were calculated from the RNAseq data, and then compared their dissimilarities (expDists) among developmental stages of different species. In calculating dissimilarity (1 – Spearman) of ortholog-group-based whole-embryonic transcriptomes (expDists), phylogenetic relationship [Anj(Apj(Sp,Lv))] were taken into consideration to avoid unwanted bias arising from simple pair-wise comparisons^58^. By randomly picking-up one biological replicate sample for each developmental stage (in each species), 100 expression tables (100 biological replicate included expression table, or BRI-exp data) were created (method reported in Hu et al.^13^). We used this 100 BRI table to test statistical significance of changes in Ptop scores (Friedman test).

### Whole-mount in situ hybridization

Digoxigenin (Dig) labeled riboprobe for *pitx* gene of *A. japonica* was prepared from PCR-amplified fragments (911 bp) using following primiers: 5ʹ-GAACGATTCGCTTCCGATGC-3ʹ (forward primer), 5ʹ-TGAGACCGGCGTATTGACAC-3ʹ (reverse primer). Whole-mount in situ hybridization (WISH) was conducted following the protocol for the planktonic larvae of a stalked crinoid *Metacrinus rotundus*^59^ with some modifications. Specimens were fixed with 4% paraformaldehyde (PFA) in 0.5 M NaCl and 0.1 M 3-(N-morpholino) propanesulfonic acid (MOPS), pH 7.0 for over 1 day, and stored at –20 °C in 70% ethanol. The fixed specimens were washed three times with PBST (1× phosphate-buffered saline with 0.1% Tween 20), treated with 0.2 µg/mL proteinase K in PBST at 37 °C for 20 min, re-fixed with 4% PFA at 4 °C for 30 min, washed three times with PBST, and then incubated in hybridization buffer (50% formamide, 5× SSC, 100 µg/mL yeast RNA, 50 µg/mL heparin, 1% Tween 20) at 55 °C for 4–6 h. Hybridization was carried out with 0.2 µg/mL probes in the hybridization buffer at 55 °C for 5 days. The protocol after hybridization was as previously described^59^. WISH finished samples were observed and photographed under the BX-51 optical microscope (Olympus).

### Proteomic analysis

Proteins were isolated from adult feather skeleton and analyzed as previously described^36^. All organic material was removed from the skeleton by extensive washing with sodium hypochlorite followed by guanidine isothiocyanate. The skeleton was demineralized with acetic acid followed by dialysis. Both soluble and insoluble protein fractions were analyzed. Proteins were separated by sodium dodecyl sulfate–polyacrylamide gel electrophoresis. Each lane was excised into 20 equal sized segments and processed. In-gel digestion with trypsin was performed on each fraction, followed by analysis by nano Liquid chromatography–mass spectrometry (LC-MS/MS) with a Waters nanoAcquity high-performance liquid chromatography system interfaced to a ThermoFisher Q Exactive hybrid quadrupole-orbitrap mass spectrometer. The mass spectrometer was operated in a data-dependent mode. Data were used to search predicted peptides from the *A. japonica* genome using Mascot and then parsed into the Scaffold algorithm for validation and filtering, using a 95% protein identification score with at least two peptides per protein.

### Statistics and reproducibility

Alpha levels of 0.05 were regarded as statistically significant throughout the study, unless otherwise specified. Experiments were repeated multiple times to confirm the reproducibility of the data. See details for individual experiments in the “Methods” sections above.

### Reporting summary

Further information on research design is available in the Nature Research Reporting Summary linked to this article.

## Supplementary information

Supplementary Information

Description of Additional Supplementary Files

Supplementary Data 1

Supplementary Data 2

Reporting Summary

## Data Availability

Genomic sequence data and assembled genomes for the following species are available through the NCBI database at the indicated BioProject accession IDs: Brittle star genome (PRJNA182997), feather star genome (PRJNA553656), and green sea urchin genome (PRJNA553643). RNAseq data are available for the following species at the indicated BioProject accession IDs: green sea urchin RNAseq data (PRJNA554218), feather star RNAseq data (PRJNA553591), and Japanese sea cucumber RNAseq data (PRJNA553613). Cloned sequences of Hox genes of the feather star (hox1 LC462021, hox2 LC462022, hox4 LC462023, hox5 LC462024, hox7 LC462025, hox8 LC462026, hox9/10 LC462027, hox11/13a LC462028, hox11/13c LC462029) are also available through the NCBI database at the indicated nucleotide accession IDs. Assembled genomes and gene sets can also be accessed through the DRYAD database^60^ at 10.5061/dryad.rbnzs7h7n. Proteomic data are available via the ProteomeXchange with identifier PXD019526. Source data for Figs. 2a and 3b can be found in Supplementary Data 1 and Supplementary Data 2.

## Code availability

No custom or proprietary software was used in the analysis. Versions and parameters for each software package used are described in the reporting summary and elsewhere in the “Methods.”
