# Author Correction: Genomic insights of body plan transitions from bilateral to pentameral symmetry in Echinoderms

**DOI:** 10.1038/s42003-021-02005-4

**Published:** 2021-04-06

**Authors:** Yongxin Li, Akihito Omori, Rachel L. Flores, Sheri Satterfield, Christine Nguyen, Tatsuya Ota, Toko Tsurugaya, Tetsuro Ikuta, Kazuho Ikeo, Mani Kikuchi, Jason C. K. Leong, Adrian Reich, Meng Hao, Wenting Wan, Yang Dong, Yaondong Ren, Si Zhang, Tao Zeng, Masahiro Uesaka, Yui Uchida, Xueyan Li, Tomoko F. Shibata, Takahiro Bino, Kota Ogawa, Shuji Shigenobu, Mariko Kondo, Fayou Wang, Luonan Chen, Gary Wessel, Hidetoshi Saiga, R. Andrew Cameron, Brian Livingston, Cynthia Bradham, Wen Wang, Naoki Irie

**Affiliations:** 1 State Key Laboratory of Genetic Resources and Evolution, Kunming Institute of Zoology, Chinese Academy of Sciences, Kunming, China; 2 Sado Island Center for Ecological Sustainability, Niigata University, Niigata, Japan; 3 Dept. of Biological Sciences, California State Univesity, Long Beach, CA USA; 4 SOKENDAI, Kanagawa, Japan; 5 Urawa University, Saitama, Japan; 6 Japan Agency for Marine-Earth Science and Technology (JAMSTEC), Kanagawa, Japan; 7 Tokyo Metropolitan University, Yokosuka, Tokyo, Japan; 8 Hokkaido University, Sapporo, Hokkaido, Japan; 9 Dept. of Biological Sciences, Graduate School of Science, The University of Tokyo, Tokyo, Japan; 10 Providence Institute of Molecular Oogenesis, Brown University, Providence, RI USA; 11 Yunnan Agricultural University, Kunming, China; 12 Shanghai Institute of Biochemistry and Cell Biology, Center for Excellence in Molecular Cell Science, Chinese Academy of Sciences, Shanghai, China; 13 RIKEN Center for Biosystems Dynamics Research (BDR), Kobe, Hyogo Japan; 14 Universal Biology Institute, University of Tokyo, Tokyo, Japan; 15 NIBB Core Research Facilities, National Institute of Basic Biology, Okazaki, Aichi Japan; 16 Faculty of Social and Cultural Studies, Kyushu University, Fukuoka, Japan; 17 Key Laboratory of Systems Biology, Hangzhou Institute for Advanced Study, University of Chinese Academy of Sciences, Chinese Academy of Sciences, Hangzhou, China; 18 Chuo University, Tokyo, Japan; 19 Beckman Institute, Division of Biology and Biological Engineering, California Institute of Technology, Pasadena, CA USA; 20 Department of Biology, Boston Univerisity, Boston, MA USA; 21 School of Ecology and Environment, Northwestern Polytechnical University, Xi’an, China

**Keywords:** Evolutionary developmental biology, Evolutionary genetics

Correction to: *Communications Biology* 10.1038/s42003-020-1091-1, published online 10 July 2020.

The original version of this Article contained an error in the author affiliations.

Affiliation 2 incorrectly read ‘Faculty of Agriculture, Niigata University, Niigata, Japan’. The correct affiliation is ‘Sado Island Center for Ecological Sustainability, Niigata University, Niigata, Japan.’

This has now been corrected both the PDF and HTML versions of the Article.

