# Supplementary material for: Genomic insights of body plan transitions from bilateral to pentameral symmetry in Echinoderms
Source: Commun Biol. 2020 Jul 10;3:371. doi: 10.1038/s42003-020-1091-1 (PMC7351957; doi:10.1038/s42003-020-1091-1)
Supplement: Supplementary file 1 — Supplementary Information [file 42003_2020_1091_MOESM1_ESM.pdf]

Supplementary Figures

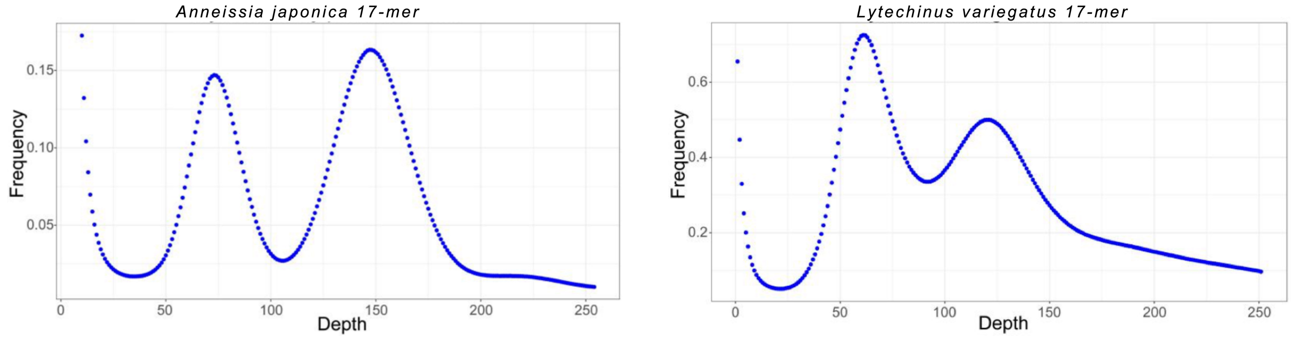

Supplementary Figure 1 Estimation of genome size by 17-mer analysis.
Kmer analysis has been done using similar method previously described[64]. The homozygous peak (around depth = 150 for *A. japonica* and 120 for *L. variegatus*) of the 17-mer frequency (M) is known to correlate with the real sequencing depth (N), read length (L), and kmer length (K), and their relations can be expressed in an experienced formula: $M = N * (L - K + 1) / L$. Estimated genome sizes of the feather star and green sea urchin were ~ 553 Mb and ~ 952 Mb respectively.

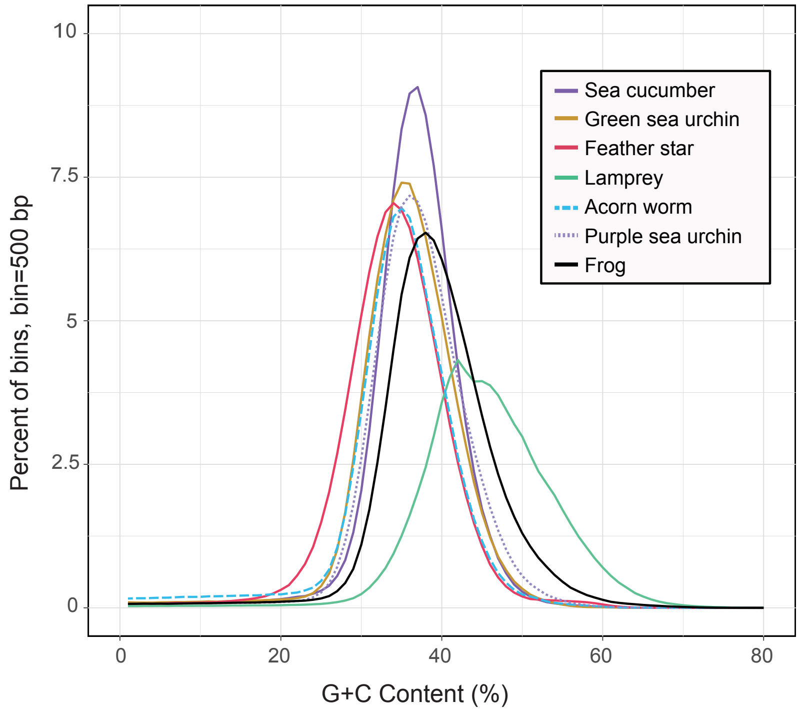

Supplementary Figure 2 GC content in genomes.
The x-axis represents GC content and the y-axis represent the proportion of the windows number divided by the total windows. The GC content was calculated using a 500bp sliding window (250 bp stepwise). Note that Feather star genome showed slightly higher G + C content compared to other echinoderms.

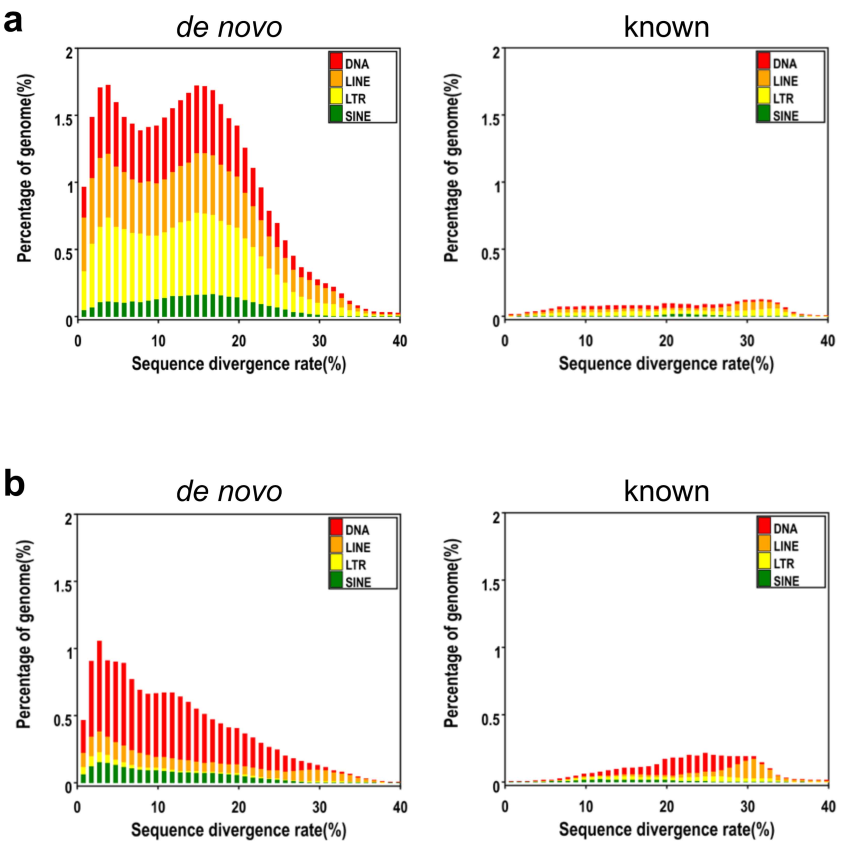

Supplementary Figure 3 The statistics of repetitive sequences.
Repetitive sequences (*de novo* predicted and known sequences) in the genomes of the feather star genome (**a**) and the green sea urchin (**b**) are shown. LINE: Long interspersed elements, LTR: Long terminal repeat, SINE: Short interspersed elements.

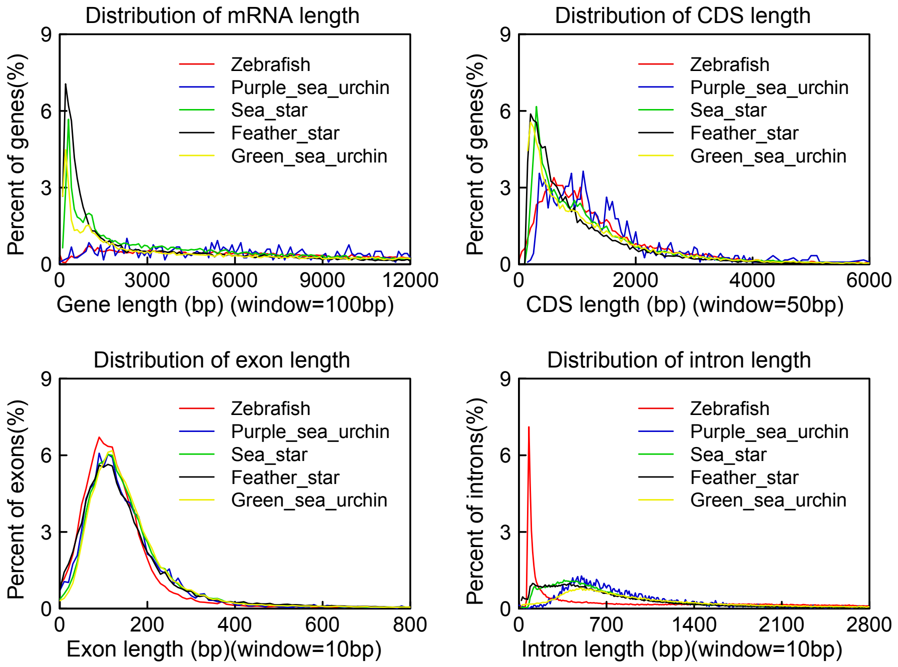

Supplementary Figure 4 Basic statistics of predicted genes for feather star and green sea urchin. Features of predicted (by Evidence Modeler, EVM) gene sets of zebrafish, purple sea urchin, sea star, feather star, and green sea urchin are illustrated. The x-axis indicates length (bp) of each genetic feature and the y-axis indicates the percentage of genes that have the corresponding length.

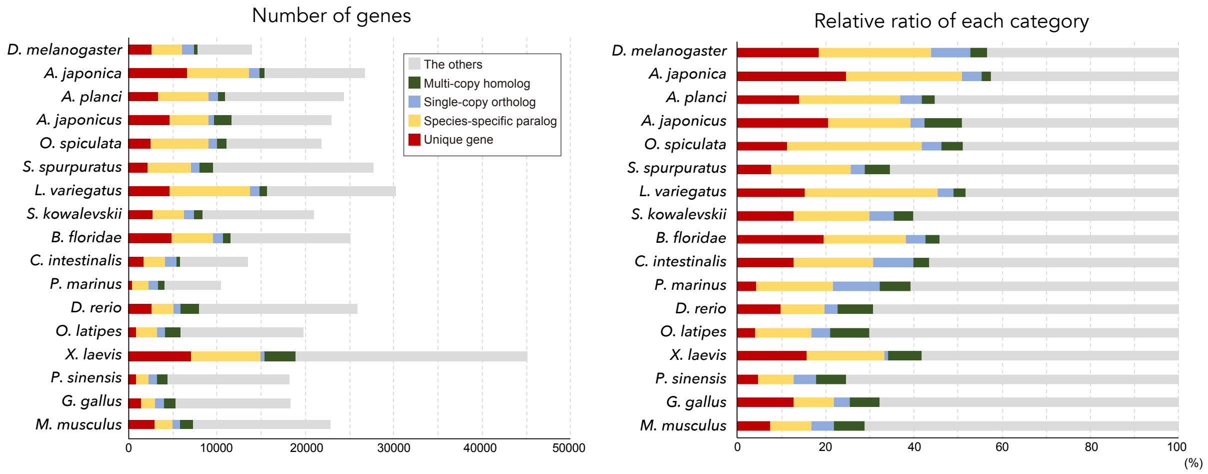

Supplementary Figure 5 The statistics of ortholog genes among 17 species.
Left panel: identified number of genes in each genome were categorized by their copy number of paralogs and homologs in the other species. Right panel: Relative ratio of categorized genes. *Unique gene* (species-specific, single copy gene): species-specific gene (no homologs in other species) without paralogs; *Species-specific paralogs*: species-specific genes (no homologs in other species) with paralogs in each species; *Single-copy ortholog*: gene that have only one copy in each species but have homologs in other species; *Multi-copy homolog*: gene that have more than one copy in each species, together with homologs in other species; *Other orthologs*: orthologs that do not belong to any type of the above orthologs/genes. The x-axis represents species used in this analysis, and y-axis represents the number of orthologs/genes in each category.

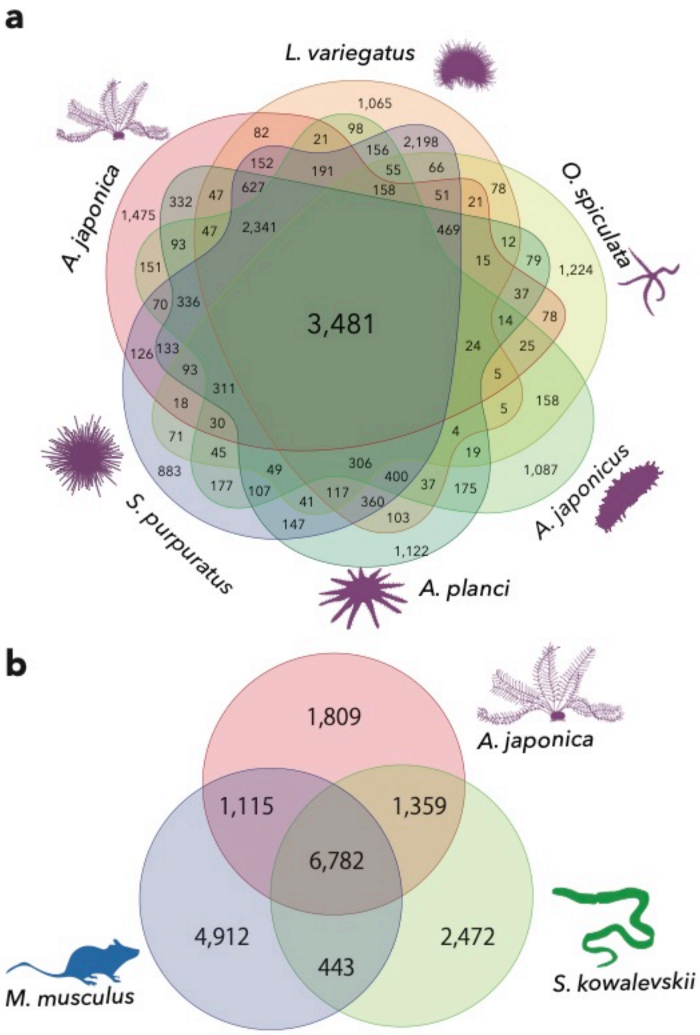

Supplementary Figure 6 Gene families shared amomng echinoderms, chordates and hemichordate
Conserved protein coding genes analyzed by analyzed by orthomcl[54]. (**a**) conserved protein coding genes among 5 echinoderm species, and (**b**) those among mouse, acon worm and feather star. Venn diagrams were drawn by R package "venn". Note that numbers indicate gene families (ortholog groups) defined by orthomcl, and do not indicate the actual numbers of coding genes.

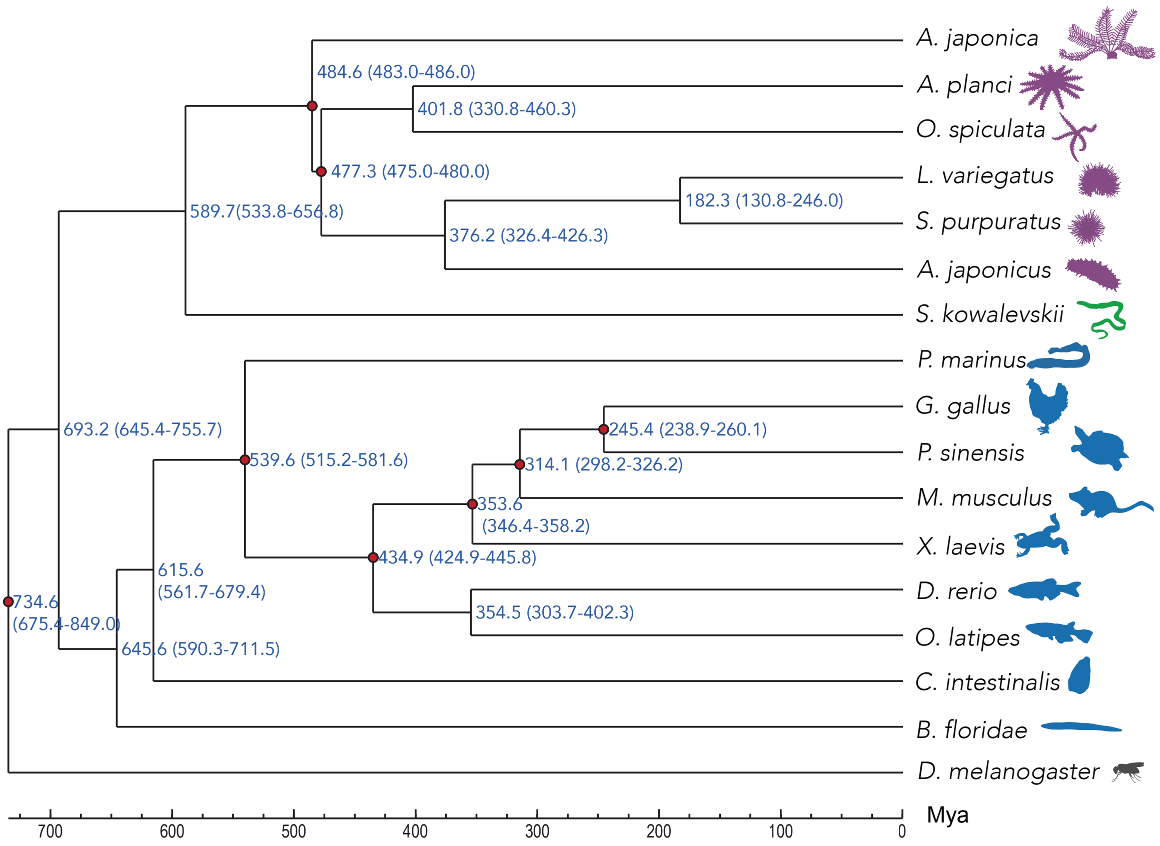

Supplementary Figure 7 Phylogeny and estimated divergence time

The tree was constructed using the 4 fold degenerate codon site of 1196 one-to-one ortholog genes. Divergence time was estimated by MCMCtree software with calibration points indicated in red points. Numbers in blue represent bootstrap values of each node.

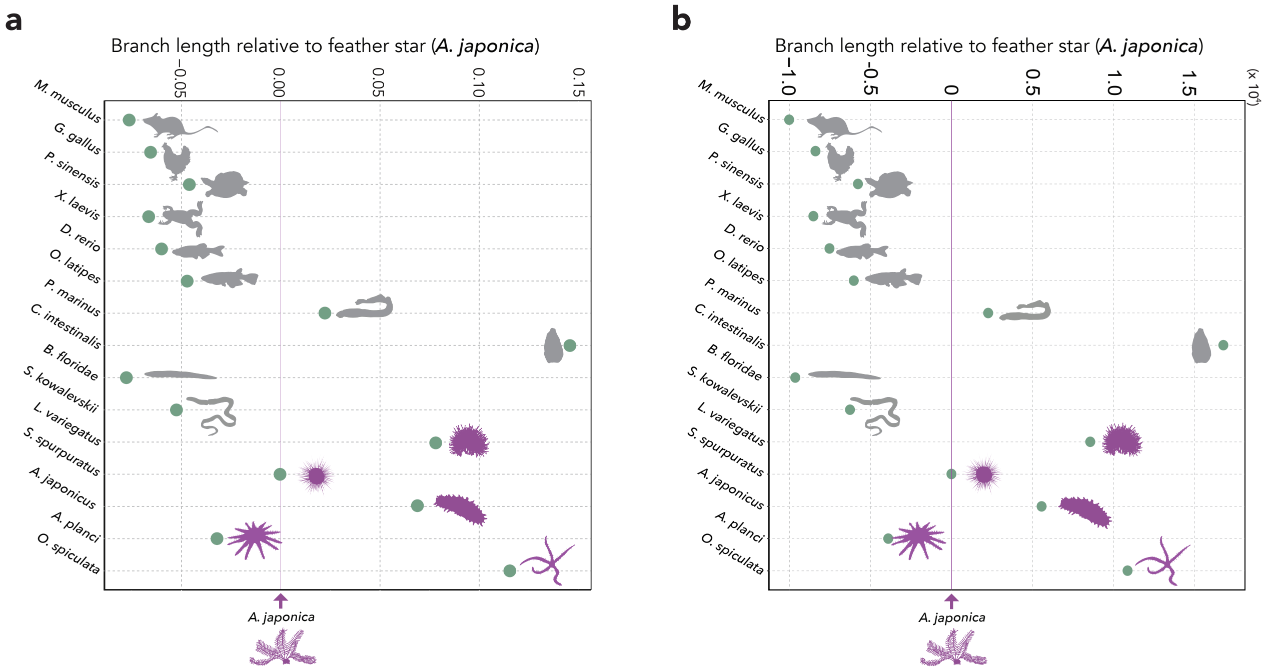

Supplementary Figure 8 Relative evolutionary rate of protein sequences

Protein sequences of 1196 one-to-one ortholog genes (identified by RBBH) were analyzed by LINTRE software (**a**) and MEGA with Tajima's Relative Ratio Test (**b**). Y axis represents relative branch length of protein sequences (compared to that of *A. japonica* from the common ancestor of echinoderms/hemichordates/chordates). While *C. intestinalis* shows the longest branch length, those of echinoderm species were relatively longer than those of most vertebrates.

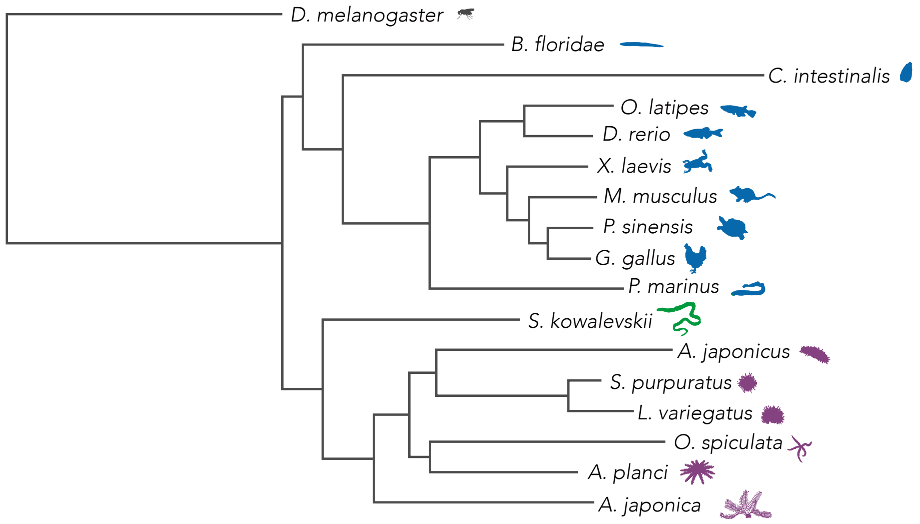

Supplementary Figure 9 Relative evolutionary rate of protein sequences analyzed by APE package. The data used in this analysis was produced with 1,196 one-to-one genes (identified by RBBH) and analyzed by APE R-package.

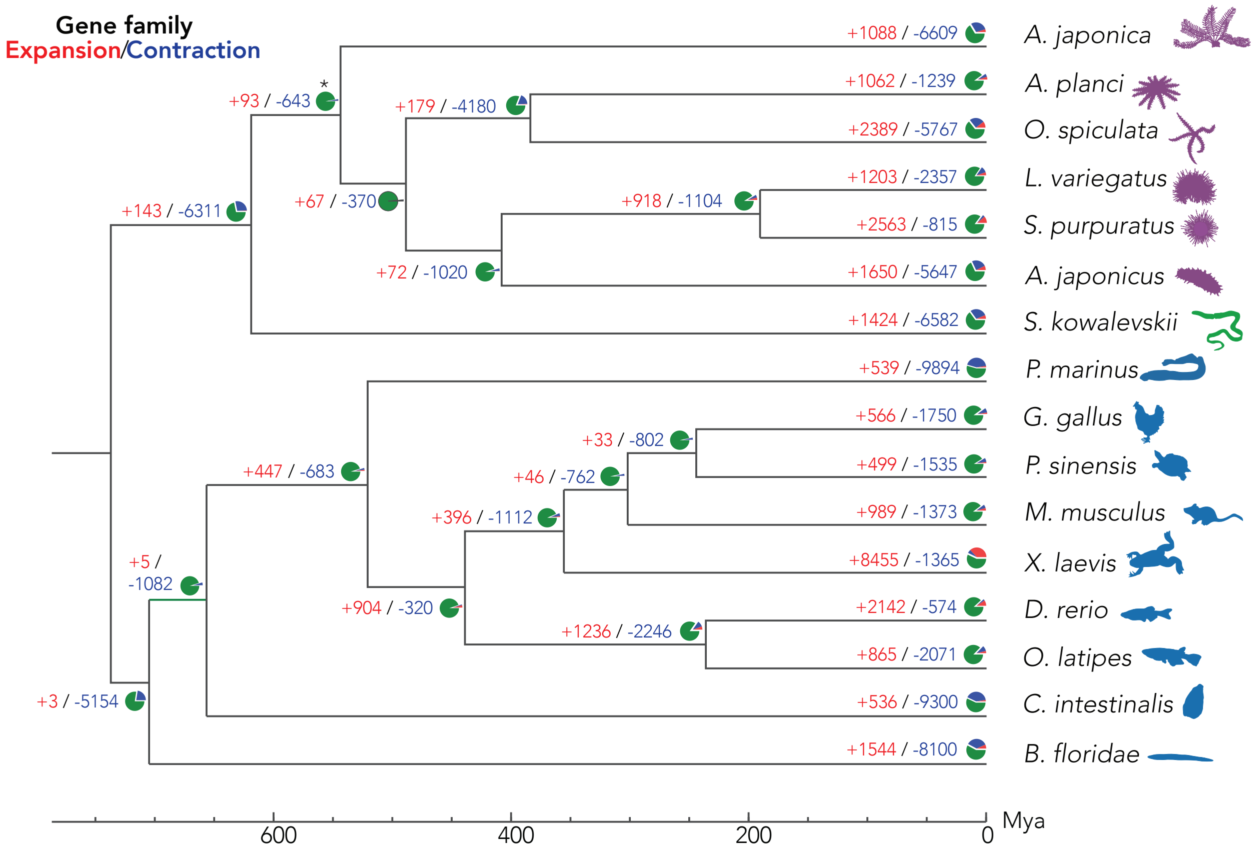

Supplementary Figure 10 Numbers of expanded and contracted gene families
Numbers in red represent numbers of expanded gene families (ortholog groups) defined by orthomcl, and numbers in blue represent those contracted gene families (ortholog groups). The pie figures on each node were drawn according to the ratio of expanded and contracted families. See Supplementary Figure 11 for GO analyses of gene families expanded / contracted in the common ancestor of echinoderms (marked with * in the figure).

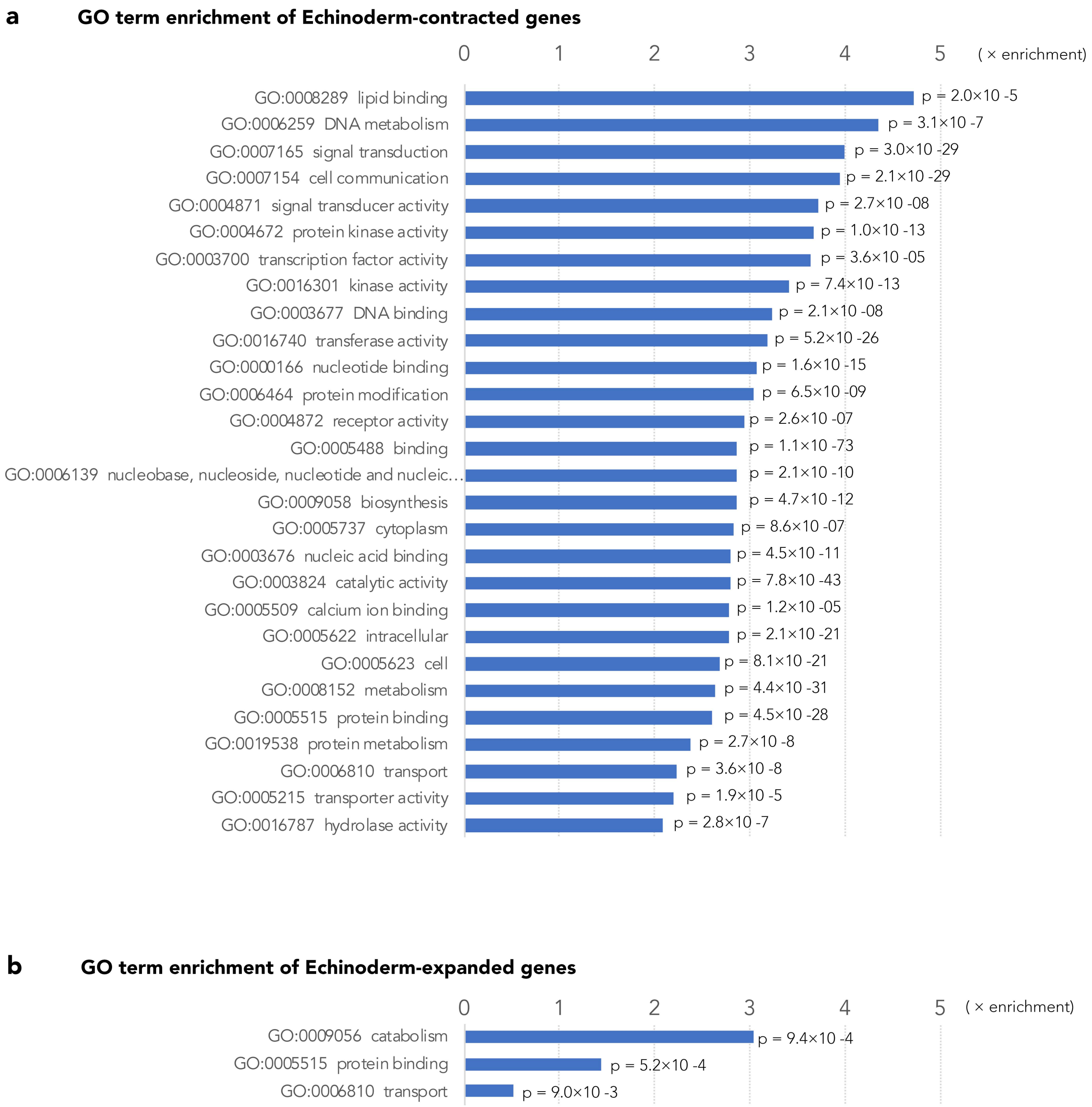

Supplementary Figure 11 GO enrichment analysis of genes expanded/contracted in echinoderm lineage Genes predicted to be contracted (**a**) and expanded (**b**) in the hypothetical common ancestor of echinoderms (see "*" position in Supplementary Figure 10 for the gene set analyzed in this figure) were analyzed for their GO term enrichment. Amphioxus (*B. floridae*) gene counterparts of the echinoderm contracted/expanded genes were first identified, and further analyzed for their GO slim term enrichment against the amphioxus genomic background using CateGOrizer (https://www.animalgenome.org/tools/catego/). Enrichment represents relative ratio of the GO terms of echinoderm-contracted/expanded genes against the genomic background. Only statistically significant results are shown here.

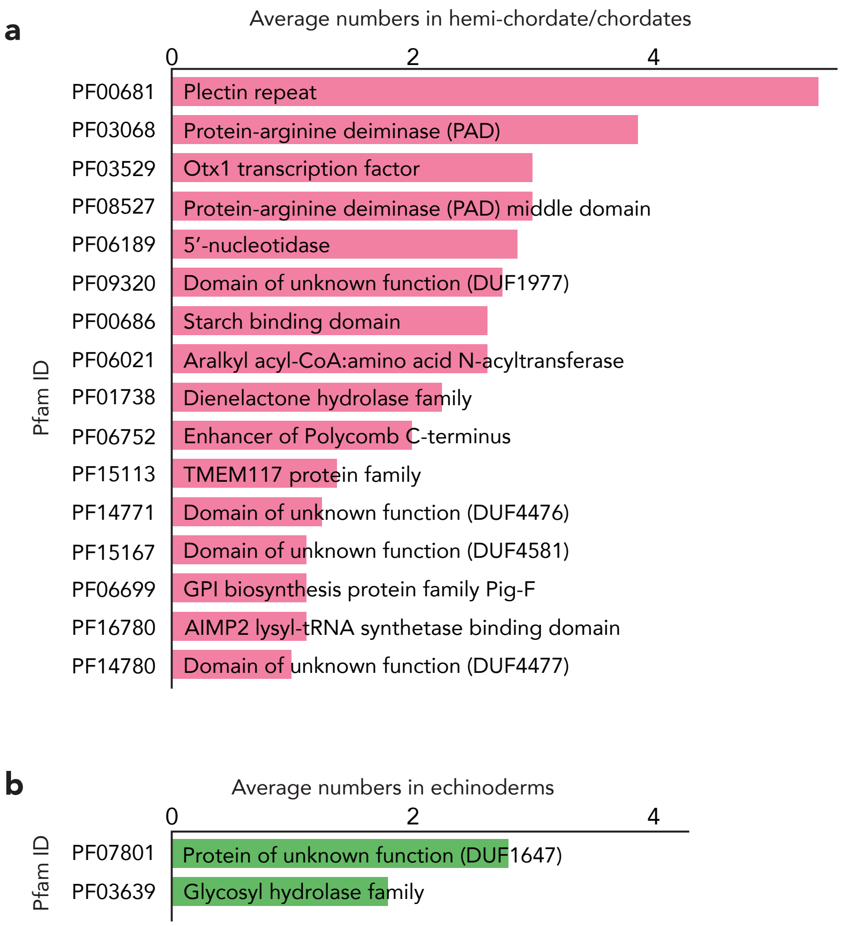

Supplementary Figure 12 Domains lost / specifically found in echinoderms
Pfam defined protein domains lost (**a**) or detected only in echinoderms (**b**) are shown. Genomes of five echinoderm species (*A. japonica*, *O. spiculata*, *L. variegatus*, *A. planci*, *A. japonicus*), and 8 other species (*S. kowalevskii*, *B. floridae*, *D. rerio*, *G. gallus*, *A. mississippiensis*, *M. musculus*, *P. marinus*, *X. laevis*) were used for this analysis. **a**. Protein domains not detected in any of the echinoderm speices, but were detected in the hemichordates or chordate species were defined as lost. **b**. Protein domains detected only in the echinoderms.

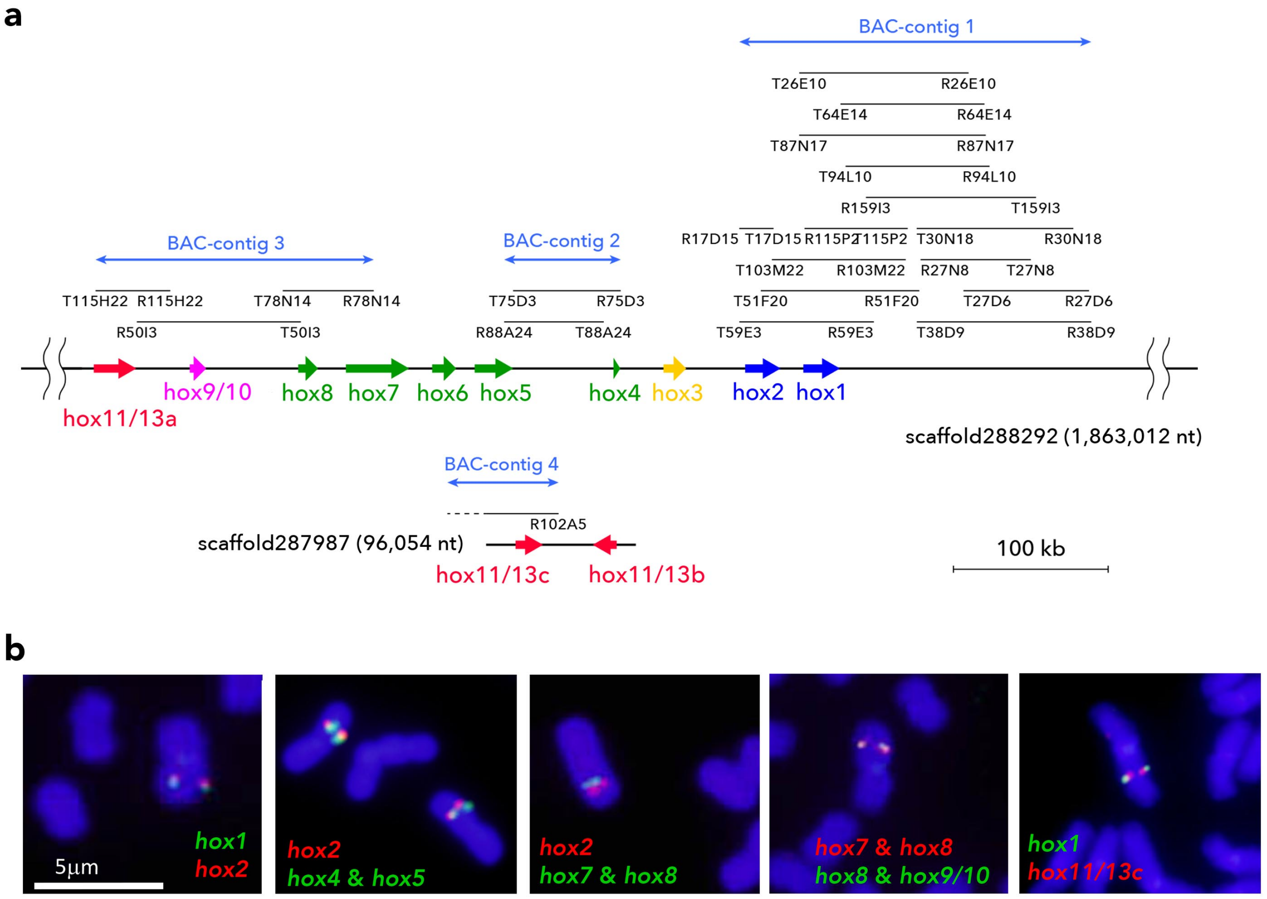

Supplementary Figure 13 Mapping of Hox genes onto scaffolds and chromosomes in feather star
a. Chromosome walking using 23 BAC clones yielded four BAC-contigs which contained hox1 and hox2 [1], hox4 and hox5 [2], hox7, hox8, hox9/10 and hox11/13a; [3] and hox11/13c [4], respectively. The 4 contigs were turned out to be included by 2 scaffolds, 288292 and 287987. **b.** Two color-chromosomal FISH. Metaphase chromosome spreads were prepared from feather star embryos and hybridized with two probes labeled with digoxigenin (red) or biotin (green) for genes indicated at the bottom of each panel. Chromosomes were stained with DAPI. Red and green spots indicate signals for the gene of the same color code. FISH analysis revealed that all of the 12 Hox genes contained in the 2 scaffolds are localized closely together on the chromosome.

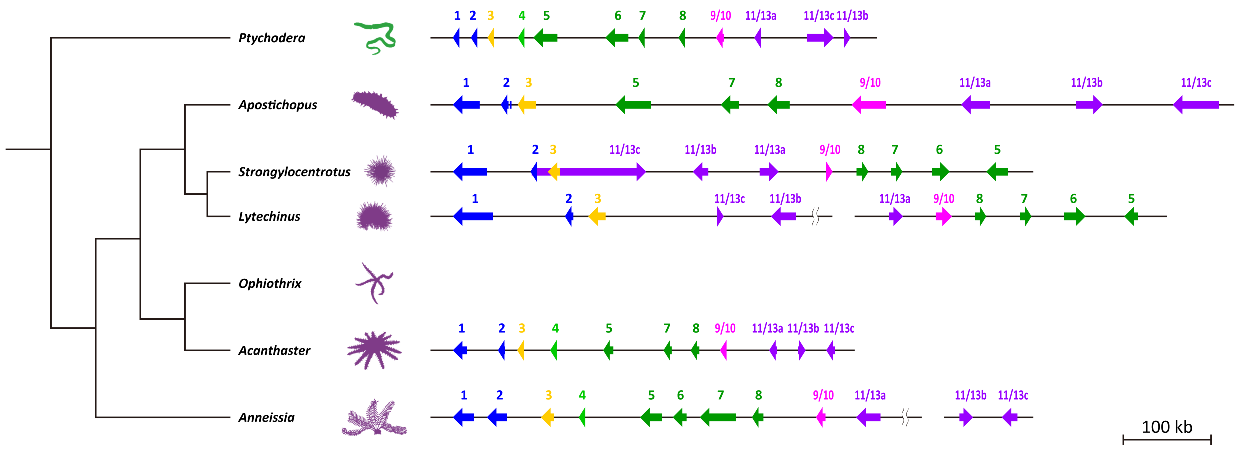

Supplementary Figure 14 Genomic organization of ambulacrarian Hox gene clusters

The ambulacrarian phylogenetic tree based on the present study and genomic organization of ambulacrarian Hox gene clusters are indicated. Arrows and solid lines represent Hox gene CDSs and scaffolds, respectively. As regards *A. japonicus hox2*, only partial CDS was identified, hence represented by the dashed arrow. In *L. variegatus*, 11 Hox genes were located on 2 scaffolds. Similarly, in *A. japonica*, 10 genes (*hox1* through *hox11/13a*) and 2 genes (*hox11/13b* and *hox11/13c*) were on 2 scaffolds, both of which were, however, on the same chromosome according to the FISH analysis (see Supplementary Fig.13). Note the (nucleotide) length from *hox11/13b* of *L. variegatus* and *hox11/13a* of *A. japonica* to the end of respective scaffolds was more than 100 kb. Previous studies were refereed for the Hox cluster structures of *P. flava*[9], *A. japonicus*[33], *S. purpuratus*[7] and *A. planci*[8].

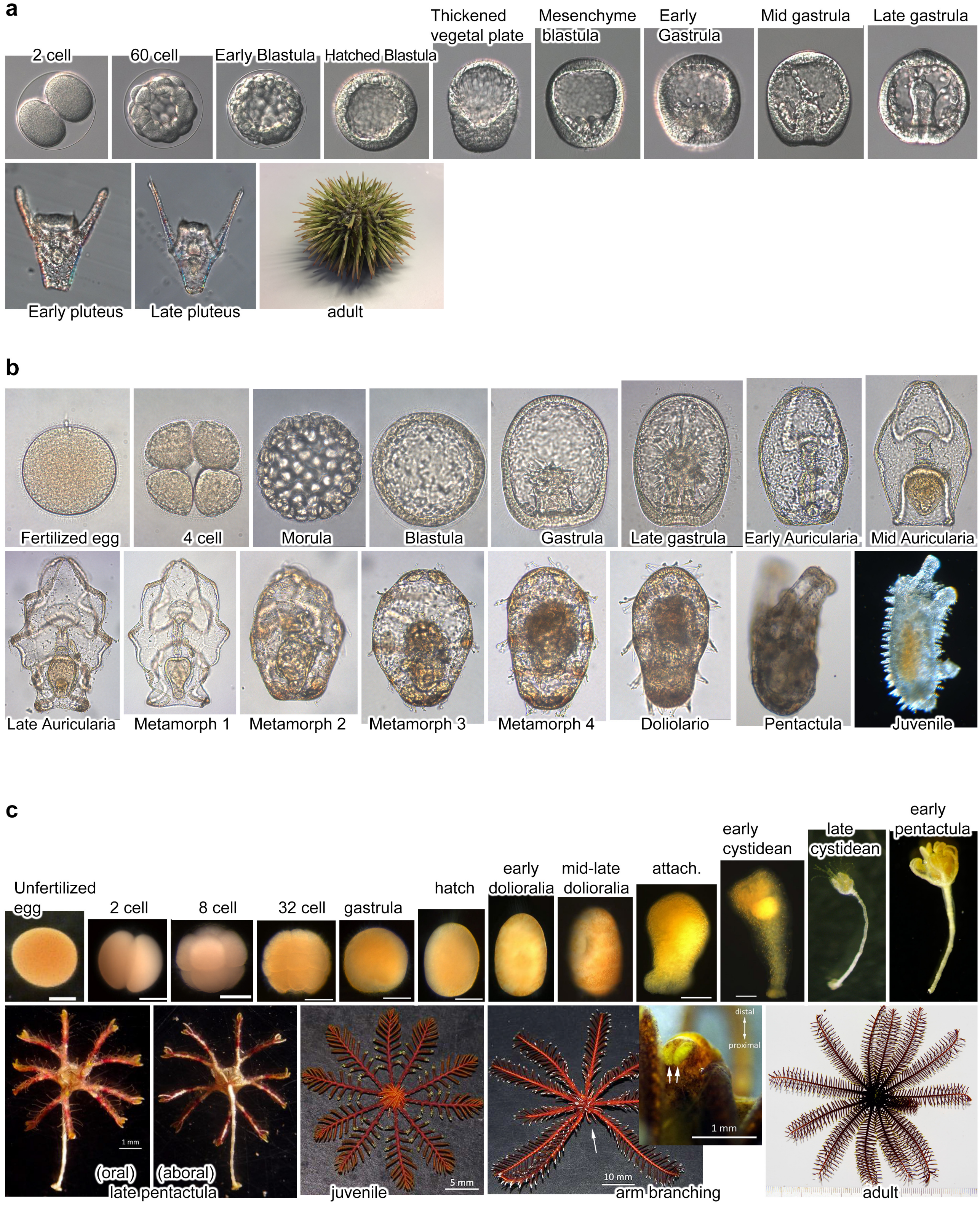

Supplementary Figure 15 Developmental stages of green sea urchin, sea cucumber and feather star
Representative images of collected echinoderm embryos for (**a**) green sea urchin (*L. variegatus*), (**b**) sea cucumber (*A. japonicus*), and (**c**) feather star (*Anneissia japonica*). (**a**) Images were modified from manuscript under preparation (Hogan *et al.*[58]). Images of the remaining stages; 7 weeks post fertilization (wpf), 8 wpf, 8 wpf Larva (without rudiment), 8 wpf rudiment, 8.1wpf, 1 day post-metamorphosis, 9wpf (1 week post-metamorphosis), are not shown here. Adult stage here corresponds to 20wpf. (**b**) Images were modified from previously published work[23]. (**c**) An arrow in the arm branching stage indicates the position of the branching arm (magnified image on it's top right). Arrows in the magnified image indicate the branching arm buds. Scale bars in unfertilized egg to early cystidean indicate 100 μm.

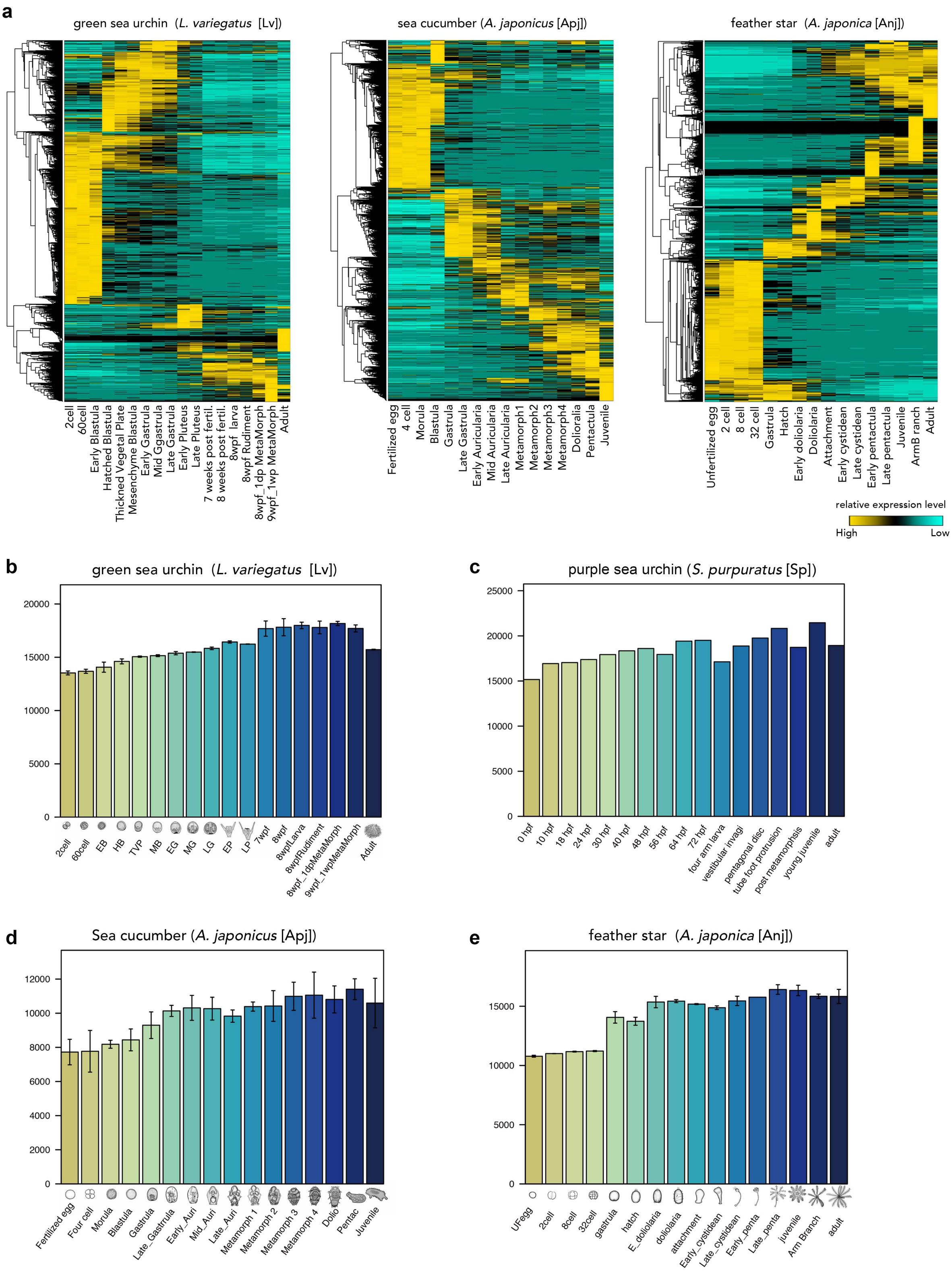

Supplementary Figure 16 Overview of gene expression patterns in echinoderm species

a. Gene expression patterns estimated from RNAseq data for each echinoderm species. For each gene, expression levels (TPM values) were z-score normalized along developmental stages. (**b-e**). Numbers of genes detected during echinoderm embryongenesis. Increasing number of coding genes detected during embryogenesis of *Lytechinus variegatus* (**b**), *Strongylocentrotus purpuratus* (**c**), *Apostichopus japonicus* (**d**), and *Anneissia japonica* (**e**). TPM higher than 1 was utilized as detection threshold, and expression levels of 10M-mapped data were used for this analysis. X-axis indicate numbers of genes detected at the stages indicated in Y-axis, and error bars indicate S.D. of numbers of detected coding genes in the biological replicate samples. Results for *S. purpuratus* do not have error bars, as the dataset (PRJNA81157) did not contain biological replicates for each stage.

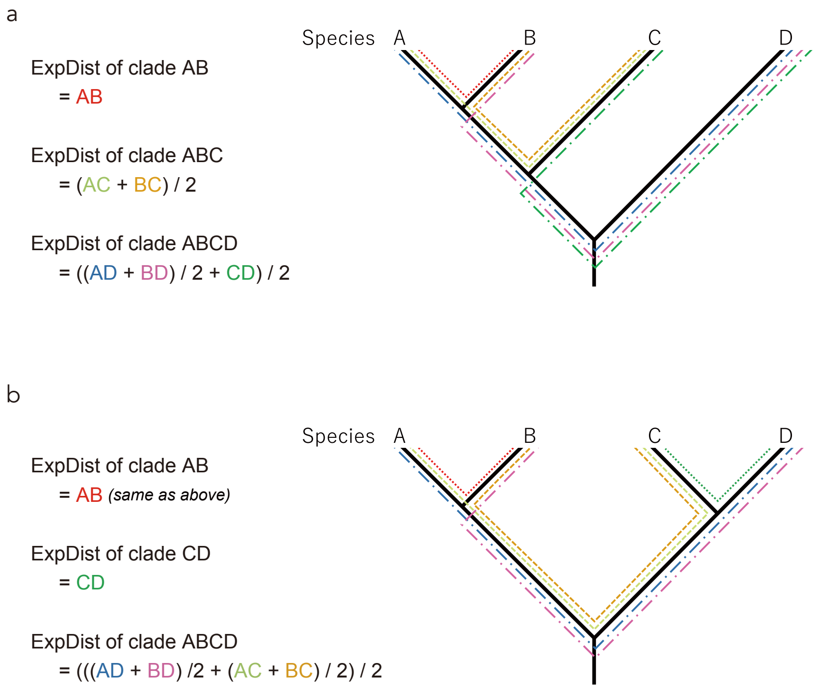

Supplementary Figure 17 Calculation of expDists for estimating conserved stages

In calculating expression Distance (expDist) of species in a single clade (as in Fig.2), phylogenetic relationship was taken into consideration to avoid unwanted bias arising from simple pair-wise comparisons of species[62]. **a**. For example, expDists in clade (A,B)C)D) was calculated as (AD + BD)/2 + CD)/2 and did not include species-pair such as BC or AC, as these do not reflect phylogenetic scale of interest. This calculation slightly differs from the previously reported method [(AD)/3 + (BD)/3 + (CD)/3 for the above case], as the previous method could be biased by distances among the species in a sub-clade (A,B)C). **b**. Hypothetical clade ((A,B),(C,D)) could be calculated as (((AD + BD)/2 + (AC + BC)/2)/2. However, this kind of phylogenetic relationship was not found in the echinoderm species we analyzed in this study, and thus was not utilized in estimating conserved stages of echinoderms.

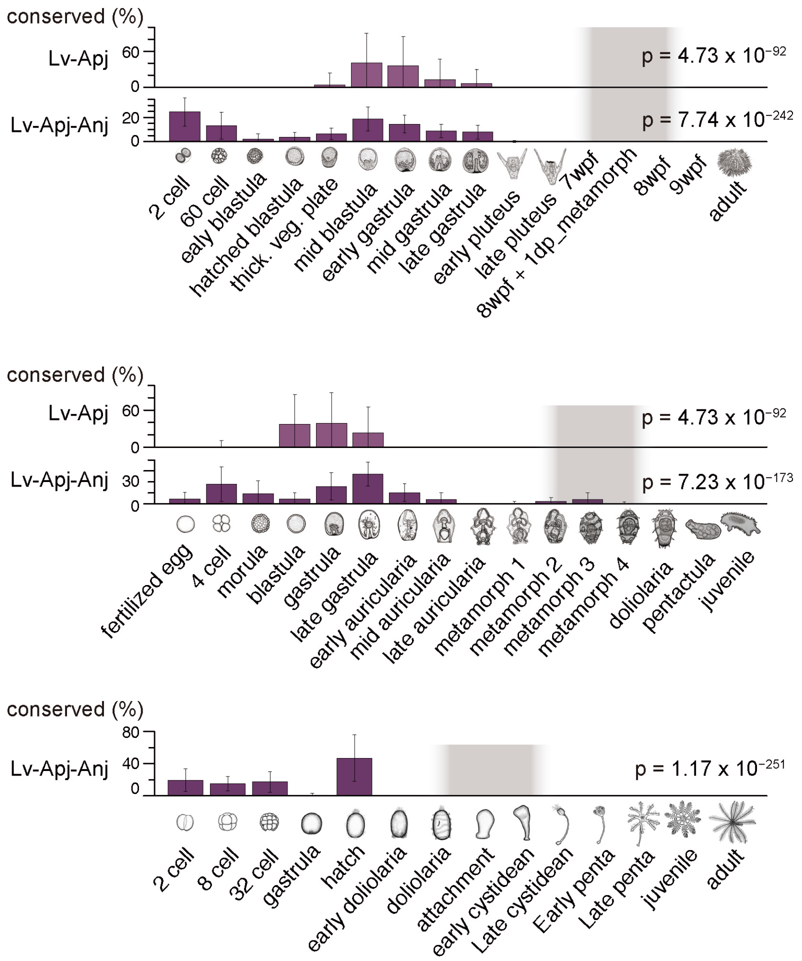

Supplementary Figure 18 Estimation of most conserved developmental stages in echinoderms without purple sea urchin.

Vertical axis represents percentages of stage being included in the most (top 1%) conserved stage-combinations[13] (Ptop). Changes of the conservation score (Ptop scores, %) were significant among stages (Friedman test). Error bars represent S.D. of Ptop values. Similar stages as in Figure 2 were found to be conserved for species without purple sea urchin (obtained from public database [PRJNA81157] sequenced by Tu Q *et al*[21,22]*)*. In each species, developmental phase when pentameral body plan establishment begins were colored in gray.

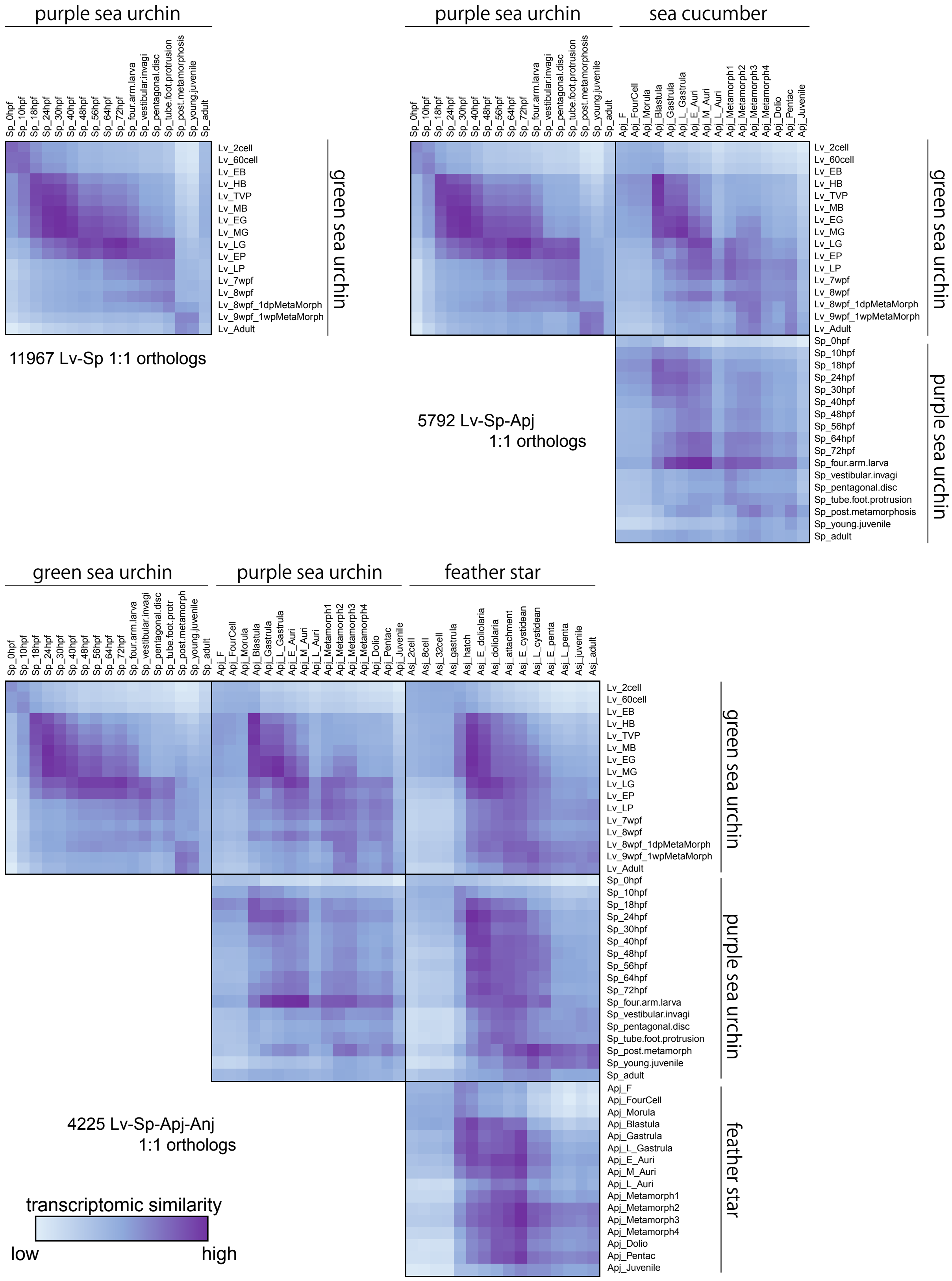

Supplementary Figure 19 Pair-wise comparisons of echinoderm transcriptome based on 1:1 orthologs
Whole embryonic transcriptome similarities were calculated for all the combinations of echinoderm stages in pair-wise manner. Expression levels (TPM) of 1:1 orthologs (defined by reciprocal best blast hits, RBBH) for Lv-Sp (11967 orthologs), Lv-Sp-Apj (5792 orthologs), and Lv-Sp-Apj-Anj (4225 orthologs) were used to calculate transcriptomic dissimilarities (1 – Spearman correlation coefficiency).

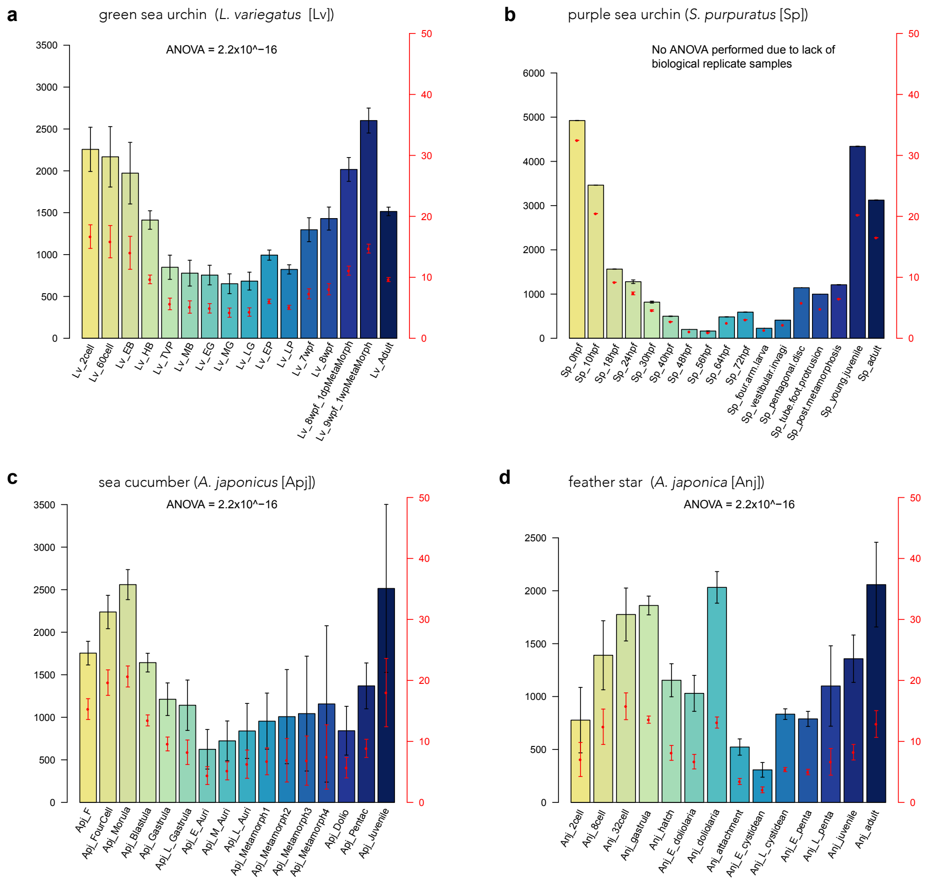

Supplementary Figure 20 Stage specific genes during echinoderm embryogenesis

Numbers of stage-specific genes were defined by tau index (tau > 0.5). Relative ratio of the stage-specific genes over the total numbers of expressed (> 1tpm) genes at each stage was also calculated (red plots, right axis in red). Error bars in panel a, c and d indicate S.D. of biological replicates calculated with the 100 random combination of expression data (BRI-exp data). The changes of these values along development were statistically significant (ANOVA without equal variance, p < 2.2 ´ 10-16).

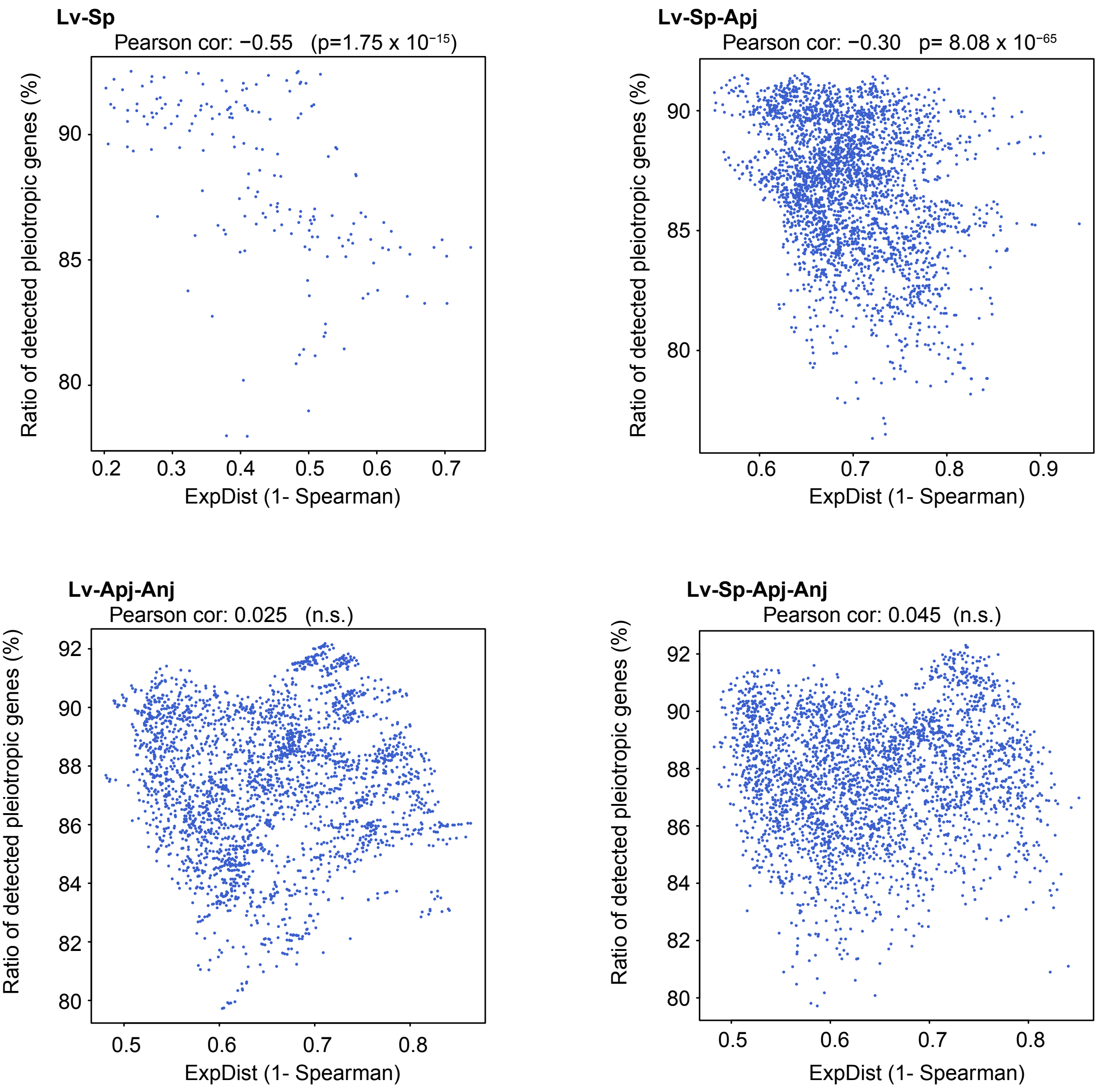

Supplementary Figure 21 Stages higher ratio of pleiotropic genes tend to be evolutionarily conserved in smaller phylogenetic group

Relationships between the ratios of pleiotropic genes (defined as genes expressed in more than 50% of staged analyzed, as in Supplementary Figure 20) and transcriptome similarity (expDsit). Each dot represent a combination of stages in the phylogenetic category of interest. For each dot, the average ratio of pleiotropic genes was calculated, and further analyzed for the relationship against their expDists (expDists). Pearson correlation coefficients and p-values (test of no correlation) are shown for each panel. Weak negative correlation was detected for Lv-Sp and Lv-Sp-Apj-Anj but not for clades of larger evolutionary scale (Lv-Apj-Anj and Lv-Sp-Apj-Anj).

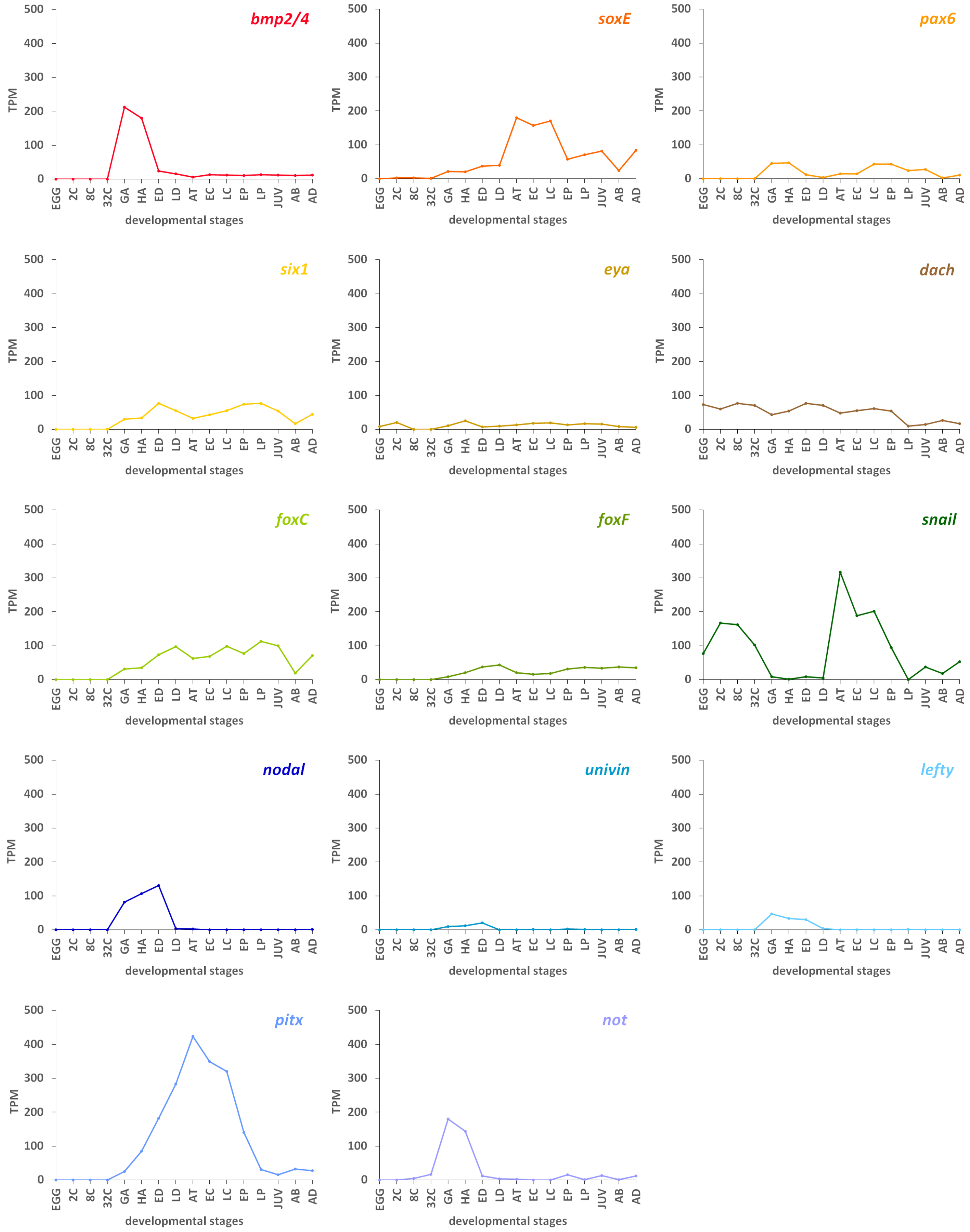

Supplementary Figure 22. Expression dynamics of left-right patterning and axis-forming genes during the feather star development.

Temporal expression profiles of the left-right (LR) patterning and axis-forming during *Anneissia japonica* embryogenesis. The horizontal and vertical axes indicate developmental stages and relative expression levels (transcripts per kilobase million, TPM, mean values of two biological replicates), respectively. Error bars are not shown according to the guideline of the journal. Abbreviations of the developmental stages indicates as follows: EGG, unfertilized egg; 2C, two cells stage; 8C, eight cells stage; 32C, 32 cells stage; GA, gastrula; HA, hatching stage; ED, early doliolaria; LD, late doliolaria; AT, attachment stage; EC, early cystidean; LC, late cystidean; EP, early pentacrinoid; LP, late pentacrinoid; JUV, juvenile; AB, arm branching stage; AD, adult. Error bars represent S.D.

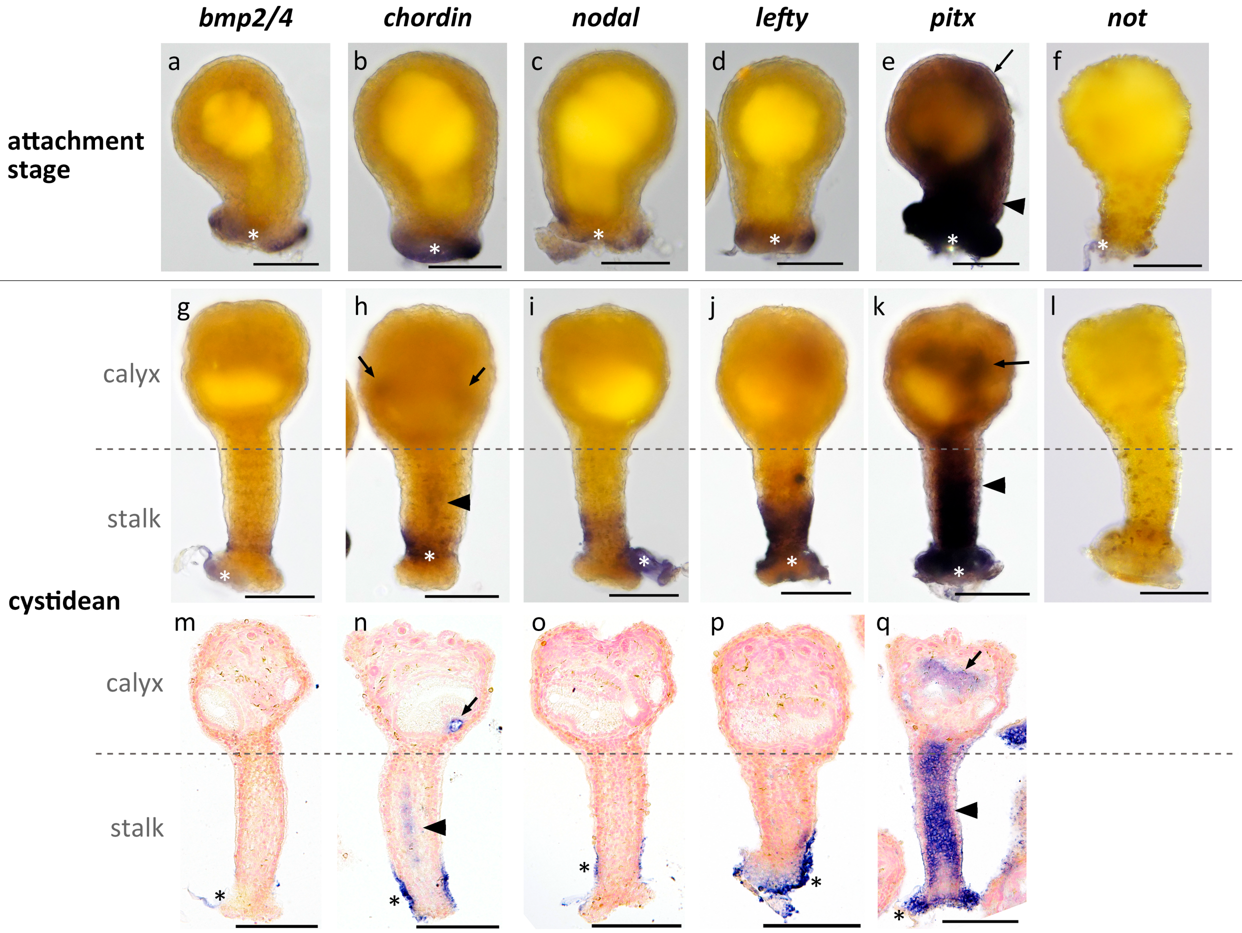

Supplementary Figure 23. Spatial expression of the left-right patterning and axis-forming in the attached larvae of feather star (*A. japonica*).
Left-right patterning related and axis-forming genes were analyzed for their expression patterns by WISH experiments. Shown are expression of *bmp2/4* (**a**, **g**, **m**), *chordin* (**b**, **h**, **n**), *nodal* (**c**, **i**, **o**), *lefty* (**d**, **j**, **p**), *pitx* (**e**, **k**, **q**), and *not* (**f**, **l**) in larvae at the attachment stage (4 days after fertilization) (**a-f**) and at the cystidean stage (6 days after fertilization) (**g-q**). Lateral views are shown for **a-l**. **m-q** are the sections of WISH samples. In the attachment stage, *pitx* was expressed in the inner tissue of the calyx (an arrow in **e**) and the forming stalk (an arrowhead in **e**), while no other genes were expressed in these tissues. In the cystidean, *chordin* was expressed in the two spots in the calyx (arrows in **h**, **n**) and the inner tissue of the stalk (arrowheads in **h**, **n**), and *pitx* was expressed in the tissues around the gut (arrows in **k, q**) and the inner tissue of the whole stalk (arrowheads in **k, q**). No other genes were expressed in the calyx. Asterisks on each figure (except **l**) indicate the staining in the outer layer of basal stalk, which may be caused by the mucus that are secreted around the attachment disk. Scale bars: 100 µm.

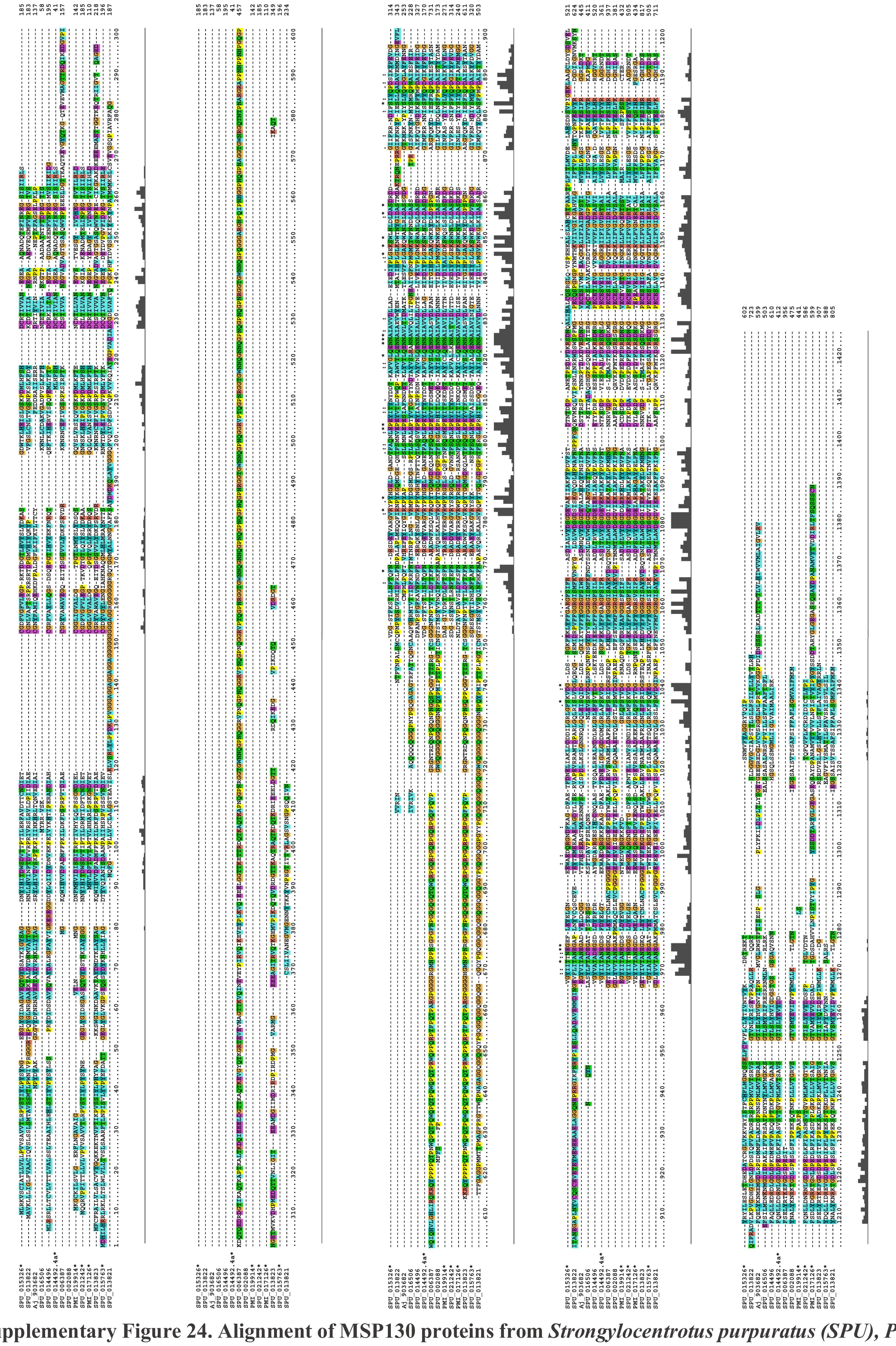

Supplementary Figure 24. Alignment of MSP130 proteins from *Strongylocentrotus purpuratus (SPU), Patiria miniata (PMI) and Oxymocanthus japonicas (Oj)*.

Proteins with a *following the name are present in the genomes but not found in the skeletal proteomes of the indicated species, while the others are found in the their respective skeletal proteomes.

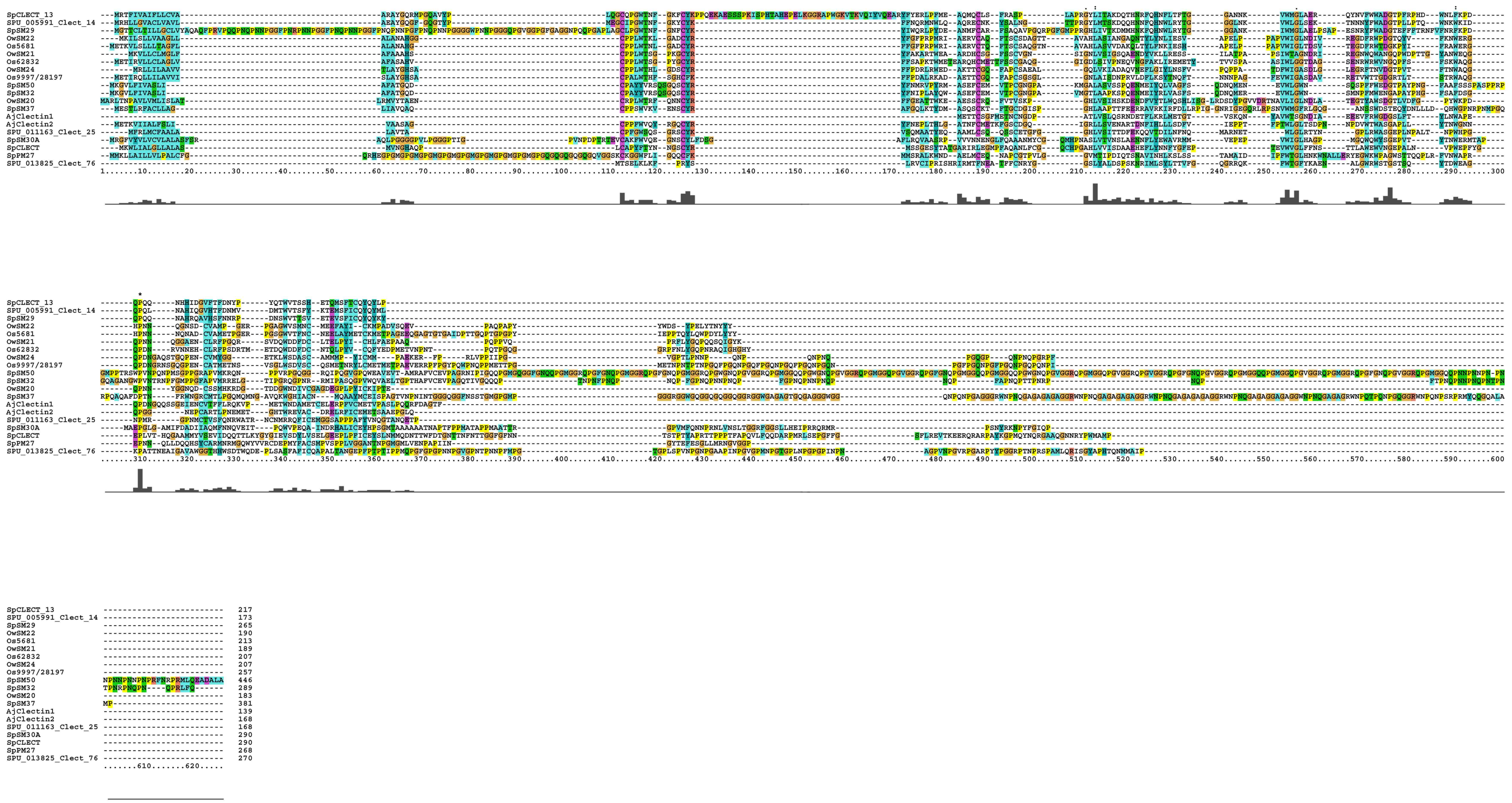

Supplementary Figure 25. Alignment of C-type lectin proteins found in the skeletal proteomes of the sea urchin *Strongylocentrotus purpuratus (SPU),* Brittle stars *Ophiocoma wendtii (Ow) and Ophothrix spiculata (Os) and* the feather star *Oxymocanthus japonicas (Oj)*.

Supplementary Tables

Library type	Library name	Insert length	Read length (bp)	Sequencing strategy	Data (Gb)	Sequencing depth (X)
Short insert library	Lv-1	394 bp	150	PE150	71.1	74.7
	Lv-2	424 bp	150	PE150	43.2	45.4
	Lv-3	479 bp	150	PE150	42.6	44.7
	Lv-4	496 bp	150	PE150	75.8	79.6
	Lv-5	522 bp	150	PE150	13.5	14.2
Long insert library	Lv-6	2-5 K	150	PE150	24.2	25.4
	Lv-7	5-9 K	150	PE150	43.7	45.9
	Lv-8	9-14 K	150	PE150	37.1	39.0
	Lv-9	14-18 K	150	PE150	29.7	31.2
		Total			380.9	400.1

Supplementary Table 1. Paired-end DNA libraries sequenced for green sea urchin assembly. Sequencing depths were calculated for high-quality clean data based on genome size (952 Mb for sea urchin). The basic statistics were performed on cleaned sequencing data.

Library type	Library name	Insert length	Read length (bp)	Sequencing strategy	Data (G)	Sequencing depth (X)
Short insert library	Anj-1	277 bp	150	PE150	32.7	59.1
	Anj -2	324 bp	150	PE150	26.9	48.6
	Anj -3	381 bp	150	PE150	14.4	26.0
	Anj -4	450 bp	150	PE150	24.2	43.8
	Anj -5	450 bp	250	PE250	36.7	66.4
	Anj -6	477 bp	150	PE150	18.8	34.0
Long insert library	Anj -7	2-5 Kb	150	PE150	6.2	11.2
	Anj -8	5-9 Kb	150	PE150	9.9	17.9
	Anj -9	14-18 Kb	150	PE150	11.0	19.9
	Anj -10	2.5 Kb	45-80	PE150	19.6	35.4
	Anj -11	3.6 Kb	45-80	PE150	7.8	14.1
	Anj -12	4.7 Kb	45-80	PE150	6.1	11.0
		Total			214.3	387.5

Supplementary Table 2. Paired-end DNA libraries sequenced for feather star genome assembly. Sequencing depths were calculated for high-quality clean data based on genome size (553Mb for feather star). The basic statistics were performed on cleaned sequencing data.

	Contig		Scaffold	
	Size (bp)	Number	Size (bp)	Number
**N90**	1,723	95,713	2,020	21,723
**N80**	3,903	60,286	24,482	2,201
**N70**	6,452	42,583	235,147	881
**N60**	8,984	30,763	434,435	582
**N50**	11,683	21,960	628,067	397
**Max length (bp)**	291126		4073095	
**Total size (bp)**	901645781		973880027	
**Total number (>100bp)**	195320		113370	
**Total number (>2000bp)**	86229		22255	

Supplementary Table 3. Basic statistics of the assembled green sea urchin genome.

	Contig		Scaffold	
	Size (bp)	Number	Size (bp)	Number
**N90**	1,478	48,708	1,805	9,910
**N80**	4,505	26,019	67,678	989
**N70**	8,618	17,091	231,513	528
**N60**	12,948	11,747	419,248	343
**N50**	18,075	8,045	623,489	228
**Max length (bp)**	198,797		5,844,021	
**Total size (bp)**	565,829,874		589,638,548	
**Total number (>100bp)**	119,876		76,733	
**Total number (>2000bp)**	40,222		8,330	

Supplementary Table 4. Basic statistics of the assembled feather star genome.

	**Contig**		**Scaffold**	
	**Size (bp)**	**Number**	**Size (bp)**	**Number**
**N90**	1,651	355,751	19,599	38,746
**N80**	2,768	253,412	32,996	28,075
**N70**	3,873	186,181	45,744	20,984
**N60**	5,082	136,373	58,599	15,651
**N50**	6,474	97,791	72,780	11,418
**Max length (bp)**	81,520		778,843	
**Total size (bp)**	2,210,140,678		2,764,315,159	
**Total number (>100bp)**	644,795		75,696	
**Total number (>2000bp)**	317,571		70,280	

Supplementary Table 5 Basic statistics of the brittle star genome obtained from Echinobase[65-67].

Library	Description	Gene number	Coverage
eukaryota_odb9	Complete BUSCOs (C)	270	89.1 %
	Complete and single-copy BUSCOs (S)	248	81.8 %
	Complete and duplicated BUSCOs (D)	22	7.3 %
	Fragmented BUSCOs (F)	14	4.6 %
	Missing BUSCOs (M)	19	6.3 %
	Total BUSCO groups searched	303	-
metazoa_odb9	Complete BUSCOs (C)	882	90.2 %
	Complete and single-copy BUSCOs (S)	815	83.3 %
	Complete and duplicated BUSCOs (D)	67	6.9 %
	Fragmented BUSCOs (F)	50	5.1 %
	Missing BUSCOs (M)	46	4.7 %
	Total BUSCO groups	978	-

Supplementary Table 6 Coverage rate of conserved genes in assembled green sea urchin genome by BUSCO.

Library	Description	Gene number	Coverage
Eukaryote-conserved genes (eukaryota_odb9)	Complete BUSCOs (C)	282	93.0 %
	Complete and single-copy BUSCOs (S)	274	90.4 %
	Complete and duplicated BUSCOs (D)	8	2.6 %
	Fragmented BUSCOs (F)	8	2.6 %
	Missing BUSCOs (M)	13	4.4 %
	Total BUSCO groups searched	303	-
Metazoa-conserved genes (metazoa_odb9)	Complete BUSCOs (C)	916	93.6 %
	Complete and single-copy BUSCOs (S)	906	92.6 %
	Complete and duplicated BUSCOs (D)	10	1.0 %
	Fragmented BUSCOs (F)	23	2.4 %
	Missing BUSCOs (M)	39	4.0 %
	Total BUSCO groups searched	978	-

Supplementary Table 7 Coverage rate of conserved genes in assembled feather star genome by BUSCO

Library	Description	Gene number	Coverage
Eukaryote-conserved genes (eukaryota_odb9)	Complete BUSCOs (C)	303	87.8 %
	Complete and single-copy BUSCOs (S)	175	57.8 %
	Complete and duplicated BUSCOs (D)	91	30.0 %
	Fragmented BUSCOs (F)	17	5.6 %
	Missing BUSCOs (M)	20	6.6 %
	Total BUSCO groups searched	303	-
Metazoa-conserved genes (metazoa_odb9)	Complete BUSCOs (C)	901	92.1 %
	Complete and single-copy BUSCOs (S)	597	61.0 %
	Complete and duplicated BUSCOs (D)	304	31.1 %
	Fragmented BUSCOs (F)	36	3.7 %
	Missing BUSCOs (M)	41	4.2 %
	Total BUSCO groups searched	978	-

Supplementary Table 8 Coverage rate of conserved genes in brittle star genome by BUSCO.

Species	Total reads	Mapped reads	Reads map (%)
Feather star	838,964,826	782,414,226	93.26
Sea urchin	1,367,973,887	1,360,681,865	99.47

Supplementary Table 9 Statistics of the mapping ratio of RNAseq reads in two assembled genomes.

Statistical item	Length (bp)	Number
N90	426	--
N80	711	--
N70	1,097	--
N60	1,568	--
N50	2,091	--
Average length	1,116	--
Max length	31,011	--
Total length	873,673,625	--
Total number	--	782,334
Number>=1000bp	--	250,683

Supplementary Table 10 Statistics of feather star transcripts assembled by Trinity and clustered by TGICL.

Statistical item	Length (bp)	Number
N90	499	--
N80	818	--
N70	1,181	--
N60	1,601	--
N50	2,063	--
Average length	1,217	--
Max length	37,771	--
Total length	239,193,964	--
Total number	--	196,425
Number>=1000bp	--	74,038

Supplementary Table 11 Statistics of green sea urchin (*Lytechinus variegatus*) transcripts assembled by Trinity and clustered by TGICL.

Range of length	Total number	Total match number	Percent (%)	>50% of sequence		>90% of sequence	
				Number	Percent(%)	Number	Percent (%)
All	453,163	447,015	98.64	430,467	94.99	320,783	70.79
>=500	227,528	225,406	99.07	217,710	95.68	162,479	71.41
>=1000	133,684	133,342	99.74	129,325	96.74	99,388	74.35

Supplementary Table 12 Assessment of the completeness of coding region using transcriptome data in the feather star genome.

Range of length (bp)	Total number	Total match number	Percent (%)	>50% of sequence		>90% of sequence	
				Number	Percent (%)	Number	Percent (%)
All	187,965	187,078	99.53	179,153	95.31	120,108	63.90
>=500	94,870	94,551	99.66	90,635	95.54	55,675	58.69
>=1000	51,093	51,057	99.93	48,655	95.23	28,087	54.97

Supplementary Table 13 Assessment of the completeness of coding region using transcriptome data in the green sea urchin genome.

Type	Repbase TEs		TE protiens		De novo		Combined TEs	
	Length (bp)	% in genome	Length (bp)	% in genome	Length (bp)	% in genome	Length (bp)	% in genome
DNA	4,329,259	0.73	2,776,494	0.47	60,982,279	10.34	65,802,851	11.16
LINE	5,273,685	0.89	20,453,430	3.47	63,607,826	10.79	86,507,741	14.67
SINE	816,335	0.14	0	0	18,668,683	3.16	18,957,279	3.22
LTR	5,897,757	1.00	7,628,210	1.30	77,710,356	13.18	88,461,182	15.00
Other	48	0.000008	0	0	14,785	0.0025	14,833	0.002
Unknown	0	0	0	0	96,699,545	16.40	96,699,545	16.40
Total	15,029,720	2.5	30,812,054	5.2	254,273,525	43.12	288,319,345	48.90

Supplementary Table 14 The statistics of transposable element in feather star genome.

Type	Repeat Size	% of genome
Trf	27,484,869	4.66
Repeatmasker	15,029,720	2.55
Proteinmask	30,812,054	5.23
De novo	270,515,276	45.88
Total	313,512,480	53.12

Supplementary Table 15 The statistics of repeat sequences annotated by different methods in feather star genome

Type	Repbase TEs		TE protiens		De novo		Combined TEs	
	Length (Bp)	% in genome	Length (Bp)	% in genome	Length (Bp)	% in genome	Length (Bp)	% in genome
DNA	15,726,621	1.61	2,700,815	0.28	104,346,168	10.71	109,578,386	11.25
LINE	10,611,419	1.09	24,815,386	2.55	26,910,567	2.76	55,762,446	5.72
SINE	2,822,575	0.29	0	0	19,535,922	2.01	20,452,191	2.10
LTR	5,661,477	0.58	5,259,001	0.54	5,808,334	0.60	16,180,259	1.66
Other	2,085	0.0002	0	0	0	0	2,085	0.0002
Unknown	0	0	0	0	253,607,055	26.04	253,607,055	26.04
Total	33,870,267	3.48	32,753,565	3.36	398,826,011	40.95	437,743,803	44.95

Supplementary Table 16 The statistics of transposable element in green sea urchin genome.

Type	Repeat Size	% of genome
Trf	41429791	4.35
Repeatmasker	33870267	3.48
Proteinmask	32753565	3.36
De novo	403226206	41.40
Total	456240421	46.84

Supplementary Table 17 The statistics of repeat sequences annotated by different methods in green sea urchin genome.

Species	Feather star	Green sea urchin
Gene number	26,838	30,238
Average mRNA length (bp)	8,836.90	16,134.76
Total number of exon	162,961	198,447
Average length of exon (bp)	191.16	203.5
Average length of CDS (bp)	1,160.73	1,335.53
Average number of exon	6.07	6.56
Total length of intron (bp)	206,013,032	447,499,073

Supplementary Table 18 The statistics of annotated gene set in feather star.

Database	Number	Percent (%)
GO	17,034	63.46
InterPro	15,389	57.34
COG	5,577	20.78
KEGG	12,146	45.25
Nr	17,880	66.62
SwissProt	14,097	52.52
TrEMBL	17,374	64.73

Supplementary Table 19 The number of gene models in feather star genome that could be annotated by GO, InterPro, COG, KEGG, Nr (RefSeq non-redundant proteins database), SwissProt and TrEMBL databases.

Database	Number	Percent (%)
GO	18,042	59.66
InterPro	16,491	54.53
COG	6,210	20.53
KEGG	13,616	45.02
Nr	20,538	67.92
SwissProt	14,962	49.48

TrEMBL	20,041	66.27

Supplementary Table 20 The number of gene models in green sea urchin genome that could be annotated by GO, InterPro, COG, KcGG, Nr, SwissProt and TrEMBL databases.

Type	Sub type	Copy (w)	Average length (bp)	Total length (bp)	% of genome
miRNA	-	330	92.60	30,559	0.0052
tRNA	-	785	73.09	57,375	0.0097
rRNA	-	365	97.33	35,524	0.0060
snRNA	-	150	146.74	22,011	0.0037
	CD-box	32	140.31	4,490	0.0008
	HACA-box	3	154	462	0.00008
	splicing	115	148.34	17,059	0.0029

Supplementary Table 21 The statistic result of ncRNAs annotation in feather star genome.

Type	Sub type	Copy(w)	Average length (bp)	Total length (bp)	% of genome
miRNA	-	595	97.84	58,213	0.0060
tRNA	-	1,131	74.45	84,206	0.0086
rRNA	-	12	334.50	4,014	0.0004
snRNA	-	187	125.89	23,542	0.0024
	Cc-box	64	101.41	6,490	0.0007
	splicing	123	138.63	17,052	0.0018

Supplementary Table 22 The statistic result of ncRNAs annotation in the green sea urchin genome.

Species	Species name	Gene count	Gene family count	Average # of genes per family
Green sea urchin	*Lytechinus variegatus*	30,238	12,877	1.65
Purple sea urchin	*Strongylocentrotus purpuratus*	27,741	13,798	1.65
Sea cucumber (*Apj*)	*Apostichopus japonicus*	21,771	10,209	1.48
Brittle star	*Ophiothrix spiculata*	22,904	7,252	2.58
Sea star	*Acanthaster planci*	24,323	11,522	1.63
Feather star (*Anj*)	*Anneissia japonica*	26,762	11,071	1.78
Acorn worm	*Saccoglossus kowalevskii*	20,935	11,101	1.56
amphioxus	*Branchiostoma belcheri*	25,135	11,167	1.84
Ciona	*Ciona intestinalis*	13,531	8,280	1.34
Lamprey	*Petromyzon marinus*	10,415	6,845	1.26
zebrafish	*Danio rerio*	25,832	13,419	1.74
Medaka	*O. lattipes*	19,699	11,934	1.44
Western clawed frog (frog)	*Xenopus laevis*	45,099	16,428	2.18
chicken	*Gallus gallus*	18,346	12,172	1.38
Chinese softcshell turtle	*Pelodiscus sinensis*	18,189	12,432	1.35
Mouse	*Mus musculus*	22,808	13,389	1.55
Fruit fly	*Drosophila melanogaster*	13,918	6,979	1.49

Supplementary Table 23 Basic statistics of genes in each species

Species name	Assembly/Gene models made by	Version information
Anolis carolinensis	Ensembl	AnoCar2.0
Branchiostoma belcheri	LanceletDB	v18h27.r3_ref
Ciona intestinalis	Ensembl	GCA_000224145.1
Danio rerio	Ensembl	GRCz10
Gallus gallus	Ensembl	Gallus_gallus-5.0
Strongylocentrotus purpuratus	NCBI	GCF_000002235.4
Saccoglossus kowalevskii	NCBI	GCF_000003605.2
Homo sapiens	Ensembl	GRCh38
Mus musculus	Ensembl	GRCm38
Petromyzon marinus	Ensembl	Pmarinus_7.0
Xenopus tropicalis	Ensembl	JGI_4.2
Drosophila melanogaster	Ensembl	BDGP6
Alligator mississippiensis	NCBI	GCF_000281125.3
Ophiothrix spiculata	Echinobase[65-67]	PRJNA182997
Apostichopus japonicus	GigaDB / NCBI	Jo J *et al.* [68] and Zhang X *et al.* [33]
Acanthaster planci	marinegenomics.oist.jp	cotsv1.0
Xenopus laevis	Xenbase	Xenla9.1_v1.8.3.2

Supplementary Table 24 Gene set and genomes used in this project.

Species	Relative branch length
A. japonica (Anj)	0.676
A. planci	0.457
O. spiculata	0.720
L. variegatus	0.198
S. purpuratus	0.099
A. japonicus (Apj)	0.726
S. kowalevskii	0.603
P. marinus	0.596
G. gallus	0.131
P. sinensis	0.142
M. musculus	0.206
X. laevis	0.245
D. rerrio	0.208
O. lattipes	0.272
C. inttestinalis	1.291
B. floridae	0.619
D. melanogaster	0.849

Supplementary Table 25 Branch length estimated from orthologous protein sequences using RAxML

Outgroup	Ingroup 1	Ingroup 2	bA	bB	delta	Z score	CP
Fruit fly	Purple sea urchin	Feather star	0.357167	0.357832	0.000664	0.248722	18.96%
Fruit fly	Sea cucumber	Feather star	0.405544	0.338478	0.067065	20.519726	99.96%
Fruit fly	Sea star (*A. planci*)	Feather star	0.325828	0.358536	0.032708	12.516316	99.96%
Fruit fly	Ciona	Feather star	0.638331	0.493120	0.145211	44.751784	99.96%
Fruit fly	Frog	Feather star	0.430825	0.497370	0.066545	23.667571	99.96%
Fruit fly	Mouse	Feather star	0.426553	0.503514	0.076962	27.491514	99.96%
Fruit fly	Amphioxus	Feather star	0.356322	0.434225	0.077903	29.249280	99.96%
Fruit fly	Acorn worm	Feather star	0.348458	0.401235	0.052777	19.409783	99.96%
Fruit fly	Turtle	Feather star	0.438257	0.484672	0.046415	16.226124	99.96%
Fruit fly	Medaka	Feather star	0.441844	0.489073	0.047229	16.736153	99.96%
Fruit fly	Zebrafish	Feather star	0.436039	0.495892	0.059853	21.198209	99.96%
Fruit fly	Chicken	Feather star	0.426225	0.491857	0.065632	23.285712	99.96%
Fruit fly	Brittle star	Feather star	0.438306	0.322925	0.115381	36.881544	99.96%
Fruit fly	Lamprey	Feather star	0.468829	0.447759	0.021071	6.902717	99.96%
Fruit fly	Green sea urchin	Feather star	0.434961	0.357281	0.077681	26.119120	99.96%

Supplementary Table 26 The result of evolutionary rate by LINTRE.
The outgroup is Drosophila, and feather star as the reference. Z-statistic was used to test whether the distances between ingroups (bA, bB) to outgroup is significantly different to 0 or not. Delta is the absolute difference between bA and bB (delta = | bA - bB |). Z-statistics(Z) is delta/standard error (Z = delta/s.e.). CP (confident probability) is equal to 1-P-value (CP= 1 - pvalue).

Outgroup	Ingroup A	Ingroup B	Identical	Ingroup A specific	Ingroup B specific	Chi-score	P-value
Fruit fly	Purple sea urchin	Feather star	249278	50403	50482	0.06	0.80358
Fruit fly	Sea cucumber	Feather star	163070	38339	32852	422.91	<0.000001
Fruit fly	Sea star (*A. planci*)	Feather star	252455	46664	50569	156.83	<0.000001
Fruit fly	Ciona	Feather star	228412	77874	61072	2031.78	<0.000001
Fruit fly	Frog	Feather star	256298	60342	68865	562.21	<0.000001
Fruit fly	Mouse	Feather star	258718	60113	70056	759.50	<0.000001
Fruit fly	Amphioxus	Feather star	242897	50022	59741	860.57	<0.000001
Fruit fly	Acorn worm	Feather star	239633	48178	54403	377.76	<0.000001
Fruit fly	Turtle	Feather star	244650	59439	65172	263.76	<0.000001
Fruit fly	Medaka	Feather star	247908	60186	66140	280.62	<0.000001
Fruit fly	Zebrafish	Feather star	253733	60393	68000	450.70	<0.000001
Fruit fly	Chicken	Feather star	251967	59428	67747	544.18	<0.000001
Fruit fly	Brittle star	Feather star	193593	49011	38062	1376.78	<0.000001
Fruit fly	Lamprey	Feather star	201026	53043	50818	47.67	<0.000001
Fruit fly	Green sea urchin	Feather star	231579	57268	48742	685.72	<0.000001

Supplementary Table 27 The result of evolutionary rate by RRT (Tajima's Relative Rate Test)

	Anj	*Ap*	*Os*	*Lv*	*Sp*	*Apj*	*Sk*	*Pm*	*Gg*	*Ps*	*Mm*	*Xl*	*Dr*	*Ol*	*Ci*	*Bf*	*Dm*
Anj	0.00	1.31	1.57	1.47	1.37	1.59	1.43	2.00	1.90	1.91	1.92	1.89	1.91	1.97	2.43	1.64	2.65
Ap	1.31	0.00	1.18	1.21	1.11	1.33	1.39	1.96	1.85	1.87	1.87	1.84	1.86	1.92	2.39	1.59	2.61
Os	1.57	1.18	0.00	1.47	1.37	1.59	1.65	2.22	2.12	2.13	2.13	2.11	2.12	2.19	2.65	1.85	2.87
Lv	1.47	1.21	1.47	0.00	0.30	1.33	1.56	2.12	2.02	2.03	2.04	2.01	2.03	2.09	2.55	1.76	2.77
Sp	1.37	1.11	1.37	0.30	0.00	1.24	1.46	2.02	1.92	1.93	1.94	1.91	1.93	1.99	2.45	1.66	2.68
Apj	1.59	1.33	1.59	1.33	1.24	0.00	1.67	2.24	2.14	2.15	2.16	2.13	2.15	2.21	2.67	1.88	2.89
Sk	1.43	1.39	1.65	1.56	1.46	1.67	0.00	1.77	1.67	1.68	1.69	1.66	1.68	1.74	2.20	1.41	2.42
Pm	2.00	1.96	2.22	2.12	2.02	2.24	1.77	0.00	1.09	1.10	1.11	1.08	1.10	1.16	2.15	1.60	2.74
Gg	1.90	1.85	2.12	2.02	1.92	2.14	1.67	1.09	0.00	0.27	0.40	0.50	0.69	0.75	2.05	1.50	2.64
Ps	1.91	1.87	2.13	2.03	1.93	2.15	1.68	1.10	0.27	0.00	0.41	0.51	0.70	0.76	2.06	1.51	2.65
Mm	1.92	1.87	2.13	2.04	1.94	2.16	1.69	1.11	0.40	0.41	0.00	0.52	0.70	0.77	2.07	1.52	2.66
Xl	1.89	1.84	2.11	2.01	1.91	2.13	1.66	1.08	0.50	0.51	0.52	0.00	0.68	0.74	2.04	1.49	2.63
Dr	1.91	1.86	2.12	2.03	1.93	2.15	1.68	1.10	0.69	0.70	0.70	0.68	0.00	0.48	2.06	1.51	2.65
Ol	1.97	1.92	2.19	2.09	1.99	2.21	1.74	1.16	0.75	0.76	0.77	0.74	0.48	0.00	2.12	1.57	2.71
Ci	2.43	2.39	2.65	2.55	2.45	2.67	2.20	2.15	2.05	2.06	2.07	2.04	2.06	2.12	0.00	2.03	3.17
Bf	1.64	1.59	1.85	1.76	1.66	1.88	1.41	1.60	1.50	1.51	1.52	1.49	1.51	1.57	2.03	0.00	2.38
Dm	2.65	2.61	2.87	2.77	2.68	2.89	2.42	2.74	2.64	2.65	2.66	2.63	2.65	2.71	3.17	2.38	0.00

Supplementary Table 28 The result of evolutionary rate by APE.

**Gene ID**	**KO ID**	**Name**	**Definition**
scaffold288031_len342695_cov171.5	K09511	DNAJB5	DnaJ homolog subfamily B member 5
scaffold287712_len2945570_cov188.9	K10397	KIF6_9	kinesin family member 6/9
scaffold287714_len2871179_cov188.42	K03130	TAF5	transcription initiation factor TFIID subunit 5
scaffold2707_len876726_cov182.13	K12571	PAN2	PAB-dependent poly(A)-specific ribonuclease subunit 2
scaffold287743_len2803612_cov181.129	K01958	PC, pyc	pyruvate carboxylase
scaffold32248_len650992_cov182.16	K12812	UAP56, BAT1, SUB2	ATP-dependent RNA helicase UAP56/SUB2
scaffold287717_len1600092_cov198.49	K12197	CHMP1, VPS46, DID2	charged multivesicular body protein 1
scaffold522_len1101056_cov227.41	K10134	EI24	etoposide-induced 2.4 mRNA
scaffold287938_len408866_cov212.4	K08469	GPR158	G protein-coupled receptor 158
scaffold287861_len940595_cov192.24	K00924	E2.7.1.-	kinase
scaffold7731_len1735946_cov199.45	K02209	MCM5, CDC46	DNA replication licensing factor MCM5
scaffold1483_len710385_cov172.5	K08857	NEK1_4_5	NIMA (never in mitosis gene a)-related kinase 1/4/5
scaffold288275_len480233_cov166.13	K04601	CELSR2	cadherin EGF LAG seven-pass G-type receptor 2
scaffold2030_len652032_cov178.10	K03251	EIF3D	translation initiation factor 3 subunit D
scaffold287715_len1027211_cov185.10	K04560	STX1A	syntaxin 1A
scaffold1489_len2284818_cov182.8	K10364	CAPZA	capping protein (actin filament) muscle Z-line, alpha
scaffold287949_len505691_cov196.9	K10733	GINS2, PSF2	GINS complex subunit 2
scaffold11779_len1430905_cov172.34	K12035	TRIM71	tripartite motif-containing protein 71
scaffold284860_len1800451_cov186.35	K08332	VAC8	vacuolar protein 8
scaffold685_len2926416_cov193.50	K12380	RASA3	Ras GTPase-activating protein 3
scaffold288014_len389662_cov180.3	K12606	RCD1, CNOT9, CAF40	CCR4-NOT transcription complex subunit 9
scaffold287861_len940595_cov192.15	K02901	RP-L27e, RPL27	large subunit ribosomal protein L27e
scaffold287835_len3118868_cov185.83	K09871	AQP12	aquaporin-12
scaffold287706_len398110_cov188.11	K07739	ELP3, KAT9	elongator complex protein 3
scaffold287847_len925385_cov155.22	K11885	DDI1	DNA damage-inducible protein 1
scaffold4054_len1135799_cov195.15	K12796	ERBB2IP, ERBIN	erbb2-interacting protein
scaffold287704_len1198815_cov201.33	K10570	ERCC8, CKN1, CSA	DNA excision repair protein ERCC-8
scaffold287754_len4335492_cov202.56	K12829	SF3B2, SAP145, CUS1	splicing factor 3B subunit 2
scaffold287868_len458714_cov195.5	K12392	AP1B1	AP-1 complex subunit beta-1
scaffold211_len1577895_cov191.47	K10151	CCND2	G1/S-specific cyclin-D2
scaffold287719_len1884721_cov185.57	K12831	SF3B4, SAP49	splicing factor 3B subunit 4
scaffold288015_len651517_cov191.8	K04460	PPP5C	serine/threonine-protein phosphatase 5
scaffold288191_len216021_cov166.10	K02885	RP-L19e, RPL19	large subunit ribosomal protein L19e
scaffold3940_len624931_cov186.12	K04922	KCNK13	potassium channel subfamily K member 13
scaffold1594_len1068242_cov183.29	K10380	ANK	ankyrin
scaffold6083_len184754_cov179.6	K10390	TUBD	tubulin delta
scaffold275_len1219475_cov178.5	K10407	KLC	kinesin light chain
scaffold287743_len2803612_cov181.22	K07199	PRKAB	5'-AMP-activated protein kinase, regulatory beta subunit
scaffold287736_len1891122_cov214.81	K00889	PIP5K	1-phosphatidylinositol-4-phosphate 5-kinase
scaffold287909_len199859_cov185.5	K13216	PPP1R8, NIPP1	nuclear inhibitor of protein phosphatase 1
scaffold376_len1572418_cov194.32	K02989	RP-S5e, RPS5	small subunit ribosomal protein S5e
scaffold234_len1672027_cov178.46	K08826	HIPK	homeodomain interacting protein kinase

scaffold306325_len3074_cov500_single.1	K13125	NOSIP	nitric oxide synthase-interacting protein
scaffold737_len1565949_cov176.8	K08291	GRK4_5_6	G protein-coupled receptor kinase
scaffold287755_len519135_cov135.3	K11338	RUVBL2, RVB2, INO80J	RuvB-like protein 2
scaffold211_len1577895_cov191.25	K02865	RP-L10Ae, RPL10A	large subunit ribosomal protein L10Ae
scaffold3782_len1007564_cov172.50	K09402	FOXJ1	forkhead box protein J1
scaffold3934_len446420_cov169.6	K14561	IMP4	U3 small nucleolar ribonucleoprotein protein
scaffold284860_len1800451_cov186.50	K00599	METTL6	methyltransferase-like protein 6
scaffold2756_len2360902_cov194.74	K00006	GPD1	glycerol-3-phosphate dehydrogenase (NAD+)
scaffold352_len1594369_cov220.6	K10165	NOD2, CARD15	nucleotide-binding oligomerization domain-containing protein 2
scaffold287949_len505691_cov196.2	K05726	BCAR1, CAS	breast cancer anti-estrogen resistance 1
scaffold1826_len1998817_cov189.4	K10085	EDEM2	ER degradation enhancer, mannosidase alpha-like 2
scaffold288039_len1115542_cov189.4	K12815	DHX38, PRP16	pre-mRNA-splicing factor ATP-dependent RNA helicase
scaffold288221_len503873_cov180.18	K05305	FUK	fucokinase
scaffold7370_len818893_cov191.42	K04638	IFT57, HIPPI, ESRRBL1	intraflagellar transport protein 57
scaffold287579_len849171_cov184.46	K10406	KIFC2_3	kinesin family member C2/C3
scaffold287711_len853857_cov187.4	K05862	VDAC1	voltage-dependent anion channel protein 1
scaffold287990_len610157_cov183.15	K14163	EPRS	bifunctional glutamyl/prolyl-tRNA synthetase
scaffold287834_len5846547_cov211.150	K14021	BAK, BAK1	Bcl-2 homologous antagonist/killer
scaffold522_len1101056_cov227.7	K11797	PHIP, WDR11	PH-interacting protein
scaffold352_len1594369_cov220.71	K09498	CCT6	T-complex protein 1 subunit zeta
scaffold287540_len12866_cov218.1	K14717	SLC39A11, ZIP11	solute carrier family 39 (zinc transporter), member 11
scaffold483_len1788099_cov177.12	K01238	SUN	SUN family beta-glucosidase
scaffold287769_len469884_cov163.11	K07561	DPH1, dph2	2-(3-amino-3-carboxypropyl)histidine synthase
scaffold585_len4108843_cov191.75	K10631	TOPORS	E3 ubiquitin-protein ligase
scaffold287790_len634930_cov199.5	K02908	RP-L30e, RPL30	large subunit ribosomal protein L30e
scaffold1696_len571195_cov180.20	K14403	CPSF3, YSH1	cleavage and polyadenylation specificity factor subunit 3

Supplementary Table 29 KEGG annotations of positively selected genes during echinoderm evolution.

References

1. Hyman, L.H. The Invertebrates: Echinodermata (Vol. IV), (McGraw-Hill, 1955).
2. Mooi, R., David, B. & Wray, G.A. Arrays in rays: terminal addition in echinoderms and its correlation with gene expression. Evol Dev 7, 542-55 (2005).
3. Rozhnov, S.V. Symmetry of echinoderms: From initial bilaterally-asymmetric metamerism to pentaradiality. *Natural Science* **06**, 171-183 (2014).
4. Reich, A., Dunn, C., Akasaka, K. & Wessel, G. Phylogenomic analyses of Echinodermata support the sister groups of Asterozoa and Echinozoa. *PLoS One* **10**, e0119627 (2015).
5. Telford, M.J. *et al.* Phylogenomic analysis of echinoderm class relationships supports Asterozoa. *Proc Biol Sci* **281**(2014).
6. Janda, L., Damborsky, J., Rezniczek, G.A. & Wiche, G. Plectin repeats and modules: strategic cysteines and their presumed impact on cytolinker functions. *Bioessays* **23**, 1064-9 (2001).
7. Cameron, R.A. *et al.* Unusual gene order and organization of the sea urchin hox cluster. *J Exp Zool B Mol Dev Evol* **306**, 45-58 (2006).
8. Baughman, K.W. *et al.* Genomic organization of Hox and ParaHox clusters in the echinoderm, Acanthaster planci. *Genesis* **52**, 952-8 (2014).
9. Freeman, R. *et al.* Identical genomic organization of two hemichordate hox clusters. *Curr Biol* **22**, 2053-8 (2012).
10. Szabo, R. & Ferrier, D.E.K. Two more Posterior Hox genes and Hox cluster dispersal in echinoderms. *BMC Evol Biol* **18**, 203 (2018).
11. Duboule, D. Temporal colinearity and the phylotypic progression: a basis for the stability of a vertebrate Bauplan and the evolution of morphologies through heterochrony. *Development*, 135-42 (1994).
12. Irie, N. & Kuratani, S. Comparative transcriptome analysis reveals vertebrate phylotypic period during organogenesis. *Nat Commun* **2**, 248 (2011).
13. Hu, H. *et al.* Constrained vertebrate evolution by pleiotropic genes. *Nat Ecol Evol* **1**, 1722-1730 (2017).
14. Zalts, H. & Yanai, I. Developmental constraints shape the evolution of the nematode mid-developmental transition. *Nat Ecol Evol* **1**, 113 (2017).
15. Kalinka, A.T. *et al.* Gene expression divergence recapitulates the developmental hourglass model. *Nature* **468**, 811-4 (2010).
16. Xu, F. *et al.* High expression of new genes in trochophore enlightening the ontogeny and evolution of trochozoans. *Sci Rep* **6**, 34664 (2016).
17. Raff, A. *The shape of life: genes, development, and the evolution of animal form*, (University of Chicago Press, 1996).
18. Irie, N., Satoh, N. & Kuratani, S. The phylum Vertebrata: a case for zoological recognition. *Zoological Lett* **4**, 32 (2018).
19. Israel, J.W. *et al.* Comparative Developmental Transcriptomics Reveals Rewiring of a Highly Conserved Gene Regulatory Network during a Major Life History Switch in the Sea Urchin Genus Heliocidaris. *PLoS Biol* **14**, e1002391 (2016).
20. Malik, A., Gildor, T., Sher, N., Layous, M. & Ben-Tabou de-Leon, S. Parallel embryonic transcriptional programs evolve under distinct constraints and may enable morphological conservation amidst adaptation. *Dev Biol* **430**, 202-213 (2017).
21. Tu, Q., Cameron, R.A. & Davidson, E.H. Quantitative developmental transcriptomes of the sea urchin Strongylocentrotus purpuratus. *Dev Biol* **385**, 160-7 (2014).
22. Tu, Q., Cameron, R.A., Worley, K.C., Gibbs, R.A. & Davidson, E.H. Gene structure in the sea urchin Strongylocentrotus purpuratus based on transcriptome analysis. *Genome Res* **22**, 2079-87 (2012).
23. Li, Y. *et al.* Weighted gene co-expression network analysis reveals potential genes involved in early metamorphosis process in sea cucumber Apostichopus japonicus. *Biochem Biophys Res Commun* **495**, 1395-1402 (2018).

24. Uchida, Y., Uesaka, M., Yamamoto, T., Takeda, H. & Irie, N. Embryonic lethality is not sufficient to explain hourglass-like conservation of vertebrate embryos. *Evodevo* **9**, 7 (2018).
25. Irie, N. Remaining questions related to the hourglass model in vertebrate evolution. *Curr Opin Genet Dev* **45**, 103–107 (2017).
26. Sumrall, C.D. & Wray, G.A. Ontogeny in the fossil record: diversification of body plans and the evolution of "aberrant" symmetry in Paleozoic echinoderms. *Paleobiology* **33**, 149–163 (2007).
27. Molina, M.D., de Croze, N., Haillot, E. & Lepage, T. Nodal: master and commander of the dorsal-ventral and left-right axes in the sea urchin embryo. *Curr Opin Genet Dev* **23**, 445–53 (2013).
28. Su, Y.H. Telling left from right: left-right asymmetric controls in sea urchins. *Genesis* **52**, 269–78 (2014).
29. Bessodes, N. *et al.* Reciprocal signaling between the ectoderm and a mesendodermal left-right organizer directs left-right determination in the sea urchin embryo. *PLoS Genet* **8**, e1003121 (2012).
30. Duboc, V., Rottinger, E., Lapraz, F., Besnardeau, L. & Lepage, T. Left-right asymmetry in the sea urchin embryo is regulated by nodal signaling on the right side. *Dev Cell* **9**, 147–58 (2005).
31. Luo, Y.J. & Su, Y.H. Opposing nodal and BMP signals regulate left-right asymmetry in the sea urchin larva. *PLoS Biol* **10**, e1001402 (2012).
32. Seaver, R.W. & Livingston, B.T. Examination of the skeletal proteome of the brittle star Ophiocoma wendtii reveals overall conservation of proteins but variation in spicule matrix proteins. *Proteome Sci* **13**, 7 (2015).
33. Zhang, X. *et al.* The sea cucumber genome provides insights into morphological evolution and visceral regeneration. *PLoS Biol* **15**, e2003790 (2017).
34. Tabari., E. & Su., Z. PorthoMCL: Parallel orthology prediction using MCL for the realm of massive genome availability. *Big Data Analytics* **2:4**(2017).
35. Sumrall, C.D. & Gregory, A.W. Ontogeny in the fossil record: Diversification of body plans and the evolution of "aberrant" symmetry in Paleozoic echinoderms. *Paleobiology* **33**, 149–163 (2007).
36. Mann, K., Wilt, F.H. & Poustka, A.J. Proteomic analysis of sea urchin (Strongylocentrotus purpuratus) spicule matrix. *Proteome Sci* **8**, 33 (2010).
37. Mann, K., Poustka, A.J. & Mann, M. In-depth, high-accuracy proteomics of sea urchin tooth organic matrix. *Proteome Sci* **6**, 33 (2008).
38. Mann, K., Poustka, A.J. & Mann, M. The sea urchin (Strongylocentrotus purpuratus) test and spine proteomes. *Proteome Sci* **6**, 22 (2008).
39. Flores, R.L. & Livingston, B.T. The skeletal proteome of the sea star Patiria miniata and evolution of biomineralization in echinoderms. *BMC Evol Biol* **17**, 125 (2017).
40. Flores, R.L., Gonzales, K., Seaver, R.W. & Livingston, B.T. The skeletal proteome of the brittle star Ophiothrix spiculata identifies C-type lectins and other proteins conserved in echinoderm skeleton formation. *AIMS Molecular Science* **3**, 357–367 (2016).
41. Ettensohn, C.A. Horizontal transfer of the msp130 gene supported the evolution of metazoan biomineralization. Evol Dev 16, 139–48 (2014).
42. Szabo, R and Ferrier, DEK. Another biomineralising protostome with an msp130 gene and conservation of msp130 gene structure across Bilateria Evolution and Development 17(3), 195–197. (2015)
43. Livingston, B.T. et al. A genome-wide analysis of biomineralization-related proteins in the sea urchin Strongylocentrotus purpuratus. Dev Biol 300, 335–48 (2006).Bedell, J.A., Korf, I. & Gish, W. MaskerAid: a performance enhancement to RepeatMasker. *Bioinformatics* **16**, 1040–1 (2000).
44. Benson, G. Tandem repeats finder: a program to analyze DNA sequences. *Nucleic Acids Res* **27**, 573–80 (1999).
45. Price, A.L., Jones, N.C. & Pevzner, P.A. De novo identification of repeat families in large genomes. *Bioinformatics* **21 Suppl 1**, i351–8 (2005).

46. Xu, Z. & Wang, H. LTR_FINDER: an efficient tool for the prediction of full-length LTR retrotransposons. *Nucleic Acids Res* **35**, W265-8 (2007).
47. Stanke, M. & Waack, S. Gene prediction with a hidden Markov model and a new intron submodel. *Bioinformatics* **19 Suppl 2**, ii215-25 (2003).
48. Burge, C. & Karlin, S. Prediction of complete gene structures in human genomic DNA. *J Mol Biol* **268**, 78-94 (1997).
49. Burge, C.B. & Karlin, S. Finding the genes in genomic DNA. *Curr Opin Struct Biol* **8**, 346-54 (1998).
50. Korf, I. Gene finding in novel genomes. *BMC Bioinformatics* **5**, 59 (2004).
51. Majoros, W.H., Pertea, M. & Salzberg, S.L. TigrScan and GlimmerHMM: two open source ab initio eukaryotic gene-finders. *Bioinformatics* **20**, 2878-9 (2004).
52. Kent, W.J. BLAT--the BLAST-like alignment tool. *Genome Res* **12**, 656-64 (2002).
53. Haas, B.J. *et al.* Automated eukaryotic gene structure annotation using EVidenceModeler and the Program to Assemble Spliced Alignments. *Genome Biol* **9**, R7 (2008).
54. Zdobnov, E.M. & Apweiler, R. InterProScan--an integration platform for the signature-recognition methods in InterPro. *Bioinformatics* **17**, 847-8 (2001).
55. Lowe, T.M. & Eddy, S.R. tRNAscan-SE: a program for improved detection of transfer RNA genes in genomic sequence. *Nucleic Acids Res* **25**, 955-64 (1997).
56. Li, L., Stoeckert, C.J., Jr. & Roos, D.S. OrthoMCL: identification of ortholog groups for eukaryotic genomes. *Genome Res* **13**, 2178-89 (2003).
57. Stamatakis, A. RAxML version 8: a tool for phylogenetic analysis and post-analysis of large phylogenies. *Bioinformatics* **30**, 1312-3 (2014).
58. Hogan, J.D.K., J. L..; Luo, L.; Hawkins, D. Y.; Ibn-Salem, J.; Lamba, A.; Schatzberg, D.; Piacentino, M. L.; Zuch, D. T.; Core, A. B.; Blumberg, C.; Timmermann, B.; Grau, J. H.; Speranza, E.; Andrade-Narravo, M. A.; Irie, N.; Poustka, A. J.; Bradham C. A. The Developmental Transcriptome for Lytechinus variegatus Exhibits Temporally Punctuated Gene Expression Changes. *Development* **460**, Issue 2, 139-154 (2020).
59. Summers, M.M., Messing, C.G. & Rouse, G.W. Phylogeny of Comatulidae (Echinodermata: Crinoidea: Comatulida): a new classification and an assessment of morphological characters for crinoid taxonomy. *Mol Phylogenet Evol* **80**, 319-39 (2014).
60. Kim, D., Langmead, B. & Salzberg, S.L. HISAT: a fast spliced aligner with low memory requirements. *Nat Methods* **12**, 357-60 (2015).
61. Pertea, M., Kim, D., Pertea, G.M., Leek, J.T. & Salzberg, S.L. Transcript-level expression analysis of RNA-seq experiments with HISAT, StringTie and Ballgown. *Nat Protoc* **11**, 1650-67 (2016).
62. Dunn, C.W., Zapata, F., Munro, C., Siebert, S. & Hejnol, A. Pairwise comparisons across species are problematic when analyzing functional genomic data. *Proc Natl Acad Sci U S A* **115**, E409-E417 (2018).
63. Omori, A., Akasaka, K., Kurokawa, D. & Amemiya, S. Gene expression analysis of Six3, Pax6, and Otx in the early development of the stalked crinoid Metacrinus rotundus. *Gene Expr Patterns* **11**, 48-56 (2011).
64. Dong, Y. *et al.* Sequencing and automated whole-genome optical mapping of the genome of a domestic goat (Capra hircus). *Nat Biotechnol* **31**, 135-41 (2013).
65. Jurka, J. *et al.* Repbase Update, a database of eukaryotic repetitive elements. *Cytogenetic and genome research* **110**, 462-7 (2005).
66. Kudtarkar, P. & Cameron, R.A. Echinobase: an expanding resource for echinoderm genomic information. *Database (Oxford)* **2017**(2017).
67. Cameron, R.A., Samanta, M., Yuan, A., He, D. & Davidson, E. SpBase: the sea urchin genome database and web site. *Nucleic Acids Res* **37**, D750-4 (2009).
68. Jo, J. *et al.* Draft genome of the sea cucumber Apostichopus japonicus and genetic polymorphism among color variants. *Gigascience* **6**, 1-6 (2017).
