# Supplementary material for: Genomic insights of body plan transitions from bilateral to pentameral symmetry in Echinoderms
Source: Commun Biol. 2020 Jul 10;3:371. doi: 10.1038/s42003-020-1091-1 (PMC7351957; doi:10.1038/s42003-020-1091-1)
Supplement: Supplementary file 2 — Description of Additional Supplementary Files [file 42003_2020_1091_MOESM2_ESM.pdf]

Description of Additional Supplementary Files

Supplementary Data 1. Source data for Figure 2a.

Supplementary Data 2. Source data for Figure 3b.
