# Supplementary material for: Genomic insights of body plan transitions from bilateral to pentameral symmetry in Echinoderms
Source: Commun Biol. 2020 Jul 10;3:371. doi: 10.1038/s42003-020-1091-1 (PMC7351957; doi:10.1038/s42003-020-1091-1)
Supplement: Supplementary file 5 — Reporting Summary [file 42003_2020_1091_MOESM5_ESM.pdf]

nature research

Corresponding author(s): Naoki Irie

Last updated by author(s): May 25, 2020

Reporting Summary

Nature Research wishes to improve the reproducibility of the work that we publish. This form provides structure for consistency and transparency in reporting. For further information on Nature Research policies, see Authors & Referees and the Editorial Policy Checklist.

Statistics

For all statistical analyses, confirm that the following items are present in the figure legend, table legend, main text, or Methods section.

n/a	Confirmed	
☐	☒	The exact sample size (*n*) for each experimental group/condition, given as a discrete number and unit of measurement
☐	☒	A statement on whether measurements were taken from distinct samples or whether the same sample was measured repeatedly
☐	☒	The statistical test(s) used AND whether they are one- or two-sided<br>*Only common tests should be described solely by name; describe more complex techniques in the Methods section.*
☐	☒	A description of all covariates tested
☐	☒	A description of any assumptions or corrections, such as tests of normality and adjustment for multiple comparisons
☐	☒	A full description of the statistical parameters including central tendency (e.g. means) or other basic estimates (e.g. regression coefficient) AND variation (e.g. standard deviation) or associated estimates of uncertainty (e.g. confidence intervals)
☐	☒	For null hypothesis testing, the test statistic (e.g. F, t, r) with confidence intervals, effect sizes, degrees of freedom and P value noted<br>*Give P values as exact values whenever suitable.*
☒	☐	For Bayesian analysis, information on the choice of priors and Markov chain Monte Carlo settings
☐	☒	For hierarchical and complex designs, identification of the appropriate level for tests and full reporting of outcomes
☐	☒	Estimates of effect sizes (e.g. Cohen's *d*, Pearson's *r*), indicating how they were calculated

Our web collection on statistics for biologists contains articles on many of the points above.

Software and code

Policy information about availability of computer code

Data collection	Illumina HiSeq 4000 platform
Data analysis	SOAPec (v2.03), GapCloser (v1.10), BUSCO (v2.0), Trinity (v.2.2.0), Tandem Repeat Finder 41 (v4.04), RepeatMasker42 (open-4-0-5) , RepeatModeler (v1.0.4), RepeatScout43 (version 1.0.5), LTR_FINDER44 (v1.0.5), repbase (RepBase16.02), Augustus45 (v2.5.5), GENSCAN46,47 (v1.0), GlimmerHMM49 (v3.02), EVidenceModeler (EVM, ver. 1.1), InterProScan (v4.8), blastp (v2.2.26), tRNAscan-SE 53 (v1.3) , BLASTN (v2.2.26) , INFERNAL software (v0.81), InterProScan52 (v4.5), INFERNAL software (v0.81), perl (v5.18.4), HISAT2 program (ver. 2.05), StringTie (ver. 1.3.5) , R (3.6.1), MCMCtree (v4.4), platanus assemble (v1.2.3), BWA (v0.7.12), SAMtools (v1.3.1), TGICL (version: linux), BLAT (v34) , SNAP (v2006-07-28), PASA (v2012-06-25), orthoMCL (v2.0.9), RAxML (v8.2.3), LINTRE (only one version available), CAFE (v3.1)

For manuscripts utilizing custom algorithms or software that are central to the research but not yet described in published literature, software must be made available to editors/reviewers. We strongly encourage code deposition in a community repository (e.g. GitHub). See the Nature Research guidelines for submitting code & software for further information.

Data

Policy information about availability of data

All manuscripts must include a data availability statement. This statement should provide the following information, where applicable:
- Accession codes, unique identifiers, or web links for publicly available datasets
- A list of figures that have associated raw data
- A description of any restrictions on data availability

Brittle star genome (PRJNA182997), feather star genome (PRJNA553656), green sea urchin genome (PRJNA553643), green sea urchin RNAseq data (PRJNA554218), feather star RNAseq data (PRJNA553591), and Japanese sea cucumber RNAseq data (PRJNA553613). Cloned sequences of Hox genes of the feather star (hox1 LC462021, hox2 LC462022, hox4 LC462023, hox5 LC462024, hox7 LC462025, hox8 LC462026, hox9/10 LC462027, hox11/13a LC462028, hox11/13c LC462029) are available through NCBI database. Assembled genomes and gene sets can also be available through DRYAD database (https://datadryad.org).

Field-specific reporting

Please select the one below that is the best fit for your research. If you are not sure, read the appropriate sections before making your selection.

☐ Life sciences ☐ Behavioural & social sciences ☒ Ecological, evolutionary & environmental sciences

For a reference copy of the document with all sections, see nature.com/documents/nr-reporting-summary-flat.pdf

Ecological, evolutionary & environmental sciences study design

All studies must disclose on these points even when the disclosure is negative.

Study description	Descriptive. Genomic and transcrtiptomic sequencing of echinoderm species (green sea urchin, feather star). Detection of gene expression by in situ hybridization. Proteome analysis.
Research sample	Single individual was used for each species (for genome sequencing).<br>Pooled individuals used for whole embryonic transcriptome data, with biological replicates for each stage. Biological replicates were collected from different parents to represent general population for each species. Further details are provided in the supplementary information file.
Sampling strategy	Single individual from Sagami Bay (Japanese feather star, Japanese sea cucumber), Reeftopia in Florida (Green sea urchin).<br>Random sampling for embryos (gender mixed). Further details are provided in the supplementary information file.
Data collection	Whole genome shotgun sequencing, and massively parallel sequencing by Illumina Hiseq 400 platform. Further details are provided in the supplementary information file.
Timing and spatial scale	Sample collections started 2015 October, and ended in 2017. During this period, embryonic samples were collected in the breeding seasons for species, and sequencing were done in early 2017.
Data exclusions	No data were excluded.
Reproducibility	All the experimental, and bioinformatics analyses were reproducible.
Randomization	For embryonic samples, samples were categorized by developmental stages defined by morphological features.
Blinding	No blindings were used in this study, except for developmental transcriptome data. Transcriptome data were analyzed blindly by a graduate student and performed some analyses, however, developmental stages were evident (easy to estimate) from the relationships of gene expression profiles between samples.

Did the study involve field work? ☐ Yes ☒ No

Reporting for specific materials, systems and methods

We require information from authors about some types of materials, experimental systems and methods used in many studies. Here, indicate whether each material, system or method listed is relevant to your study. If you are not sure if a list item applies to your research, read the appropriate section before selecting a response.

Materials & experimental systems

n/a	Involved in the study	
☒	☐	Antibodies
☒	☐	Eukaryotic cell lines
☒	☐	Palaeontology
☐	☒	Animals and other organisms
☒	☐	Human research participants
☒	☐	Clinical data

Methods

n/a	Involved in the study	
☒	☐	ChIP-seq
☒	☐	Flow cytometry
☒	☐	MRI-based neuroimaging

Animals and other organisms

Policy information about studies involving animals; ARRIVE guidelines recommended for reporting animal research

Laboratory animals	All the animals were collected from the wild.
Wild animals	Green sea urchin(Lytechinus variegatus): Adult green sea urchins obtained from Reeftopia in Florida (FL) or from the Duke Marine lab in Beaufort NC.<br>Japanese feather star (Anneissia japonica): in the cove of Koajiro, Sagami Bay (Misaki, Japan) by scuba diving.

	Sea cucumber (Apostichopus japonicus): collected from a subtidal sandy benthic environment in Koajiro, Misaki, Sagami Bay (Japan).
Field-collected samples	Fertilized eggs were kept in sea water with room temperature.
Ethics oversight	Animal care and experimental procedures and were conducted in strict accordance with guidelines approved by the Animal Experiments Committee of University of Tokyo (approval ID: 14-03, 16-2). All efforts were made to minimize suffering. Individual animals and embryos were selected blindly from wild types.

Note that full information on the approval of the study protocol must also be provided in the manuscript.
